# Lifestyle factors and subacromial impingement syndrome of the shoulder: potential associations in finnish participants

**DOI:** 10.1186/s12891-024-07345-w

**Published:** 2024-03-19

**Authors:** Zhengtao Lv, Jiarui Cui, Jiaming Zhang, Li He

**Affiliations:** 1 Department of Orthopedics, Tongji Hospital, Tongji Medical College, Huazhong University of Science and Technology, Wuhan, 430030 China; 2 Clinical Innovation & Research Center (CIRC), Shenzhen Hospital, Southern Medical University, Shenzhen, 518100 China; 3 Department of Traumatic Surgery, Tongji Hospital, Tongji Medical College, Huazhong University of Science and Technology, 1095#, Jie-Fang Avenue, Qiaokou District, Wuhan, 430030 China

**Keywords:** Subacromial impingement syndrome, Lifestyle factor, Risk, Causal effect, Mendelian randomization

## Abstract

**Background:**

Emerging evidence has indicated the associations between subacromial impingement syndrome (SIS) of shoulder and lifestyle factors. However, whether unhealthy lifestyle factors causally increase SIS risk is not determined. This study aims to evaluate whether lifestyle factors are the risk factors of SIS.

**Methods:**

A two-sample Mendelian randomization (MR) study was designed to evaluate the effect of 11 lifestyle factors on SIS risk. Causality was determined using the inverse-variance weighted method to calculate the odds ratio (OR) and establish a 95% confidence interval (CI). Weighted median method, MR-Egger method and MR-PRESSO method were conducted as sensitivity analysis.

**Results:**

Four lifestyle factors were identified causally associated with an increased risk of SIS using the IVW method: insomnia (OR: 1.66 95% CI 1.38, 2.00; *P* = 8.86 × 10^− 8^), short sleep duration (OR: 1.53 95% CI 1.14, 2.05: *P* = 0.0043), mobile phone usage (OR: 4.65, 95% CI 1.59, 13.64; *P* = 0.0051), and heavy manual or physical work (OR: 4.24, 95% CI 2.17, 8.26; *P* = 2.20 × 10^− 5^). Another causal but weak association was found between smoking initiation on SIS (OR: 1.17, 95% CI 1.01, 1.35; *P* = 3.50 × 10^− 2^). Alcohol, coffee consumption, physical activity, sedentary behavior, sleep duration and computer usage were not found to be causally associated with an increased risk of SIS. Sensitivity analyses indicated that the MR estimates were robust and no heterogeneity and pleiotropy were identified in these MR analyses.

**Conclusion:**

Sleep habits and shoulder usage were identified as causal factors for SIS. This evidence supports the development of strategies aimed at improving sleep behaviors and optimizing shoulder usage patterns as effective measures to prevent SIS.

**Supplementary Information:**

The online version contains supplementary material available at 10.1186/s12891-024-07345-w.

## Background

Subacromial impingement syndrome (SIS) of shoulder is a common musculoskeletal disorder in both athletic and non-athletic populations [1] and is considered the most common cause of shoulder pain [2], accounting for 36% of all shoulder disorders [3]. Common consequences of SIS include persistent pain and disability [4], which not only severely reduce life quality, such as causing sleep disorders [5], but also lead to significant consumption of medical resources and social costs [6, 7]. Therefore, there is an urgent need to identify the risk factors for the early prevention of SIS.

Identifying causal effects of unhealthy lifestyle factors on the human disease can help us develop feasible and effective strategies to reduce disease risk. An observational study suggested that SIS was associated with smoking, sleep problems and occupational mechanical exposure [8]. While, another study showed smoking and alcohol consumption didn’t involve in the natural course of SIS [9]. Meanwhile, SIS might conversely deteriorate sleep problems [5]. Cumulative occupational mechanical exposures further increased the risk of surgery for SIS [10]. Physical activity might involve repetitive overhead motion or excessive loading of the shoulder joint, while prolonged sitting with poor posture can lead to muscle imbalances and postural changes, both of which may increase the risk of SIS. On the contrary, development of SIS can limit an individual’s physical activity and increase the likelihood of sedentary behavior [11]. A systematic review concluded that exercises were effective in the management of SIS [12]. Coffee consumption, computer usage, and mobile phone usage are modern lifestyle factors. Coffee consumption was associated with increased inflammation and decreased muscle recovery [13]. But coffee consumption can alleviate shoulder pain during computer office work [14]. Prolonged computer and mobile phone usage might lead to poor posture and repetitive strain injuries, and may cause musculoskeletal disorders [15]. Another study found no indication that exposure to computer usage is related to neck and shoulder symptoms [16]. These associations might be limited to be causality by inherent disadvantages of observational studies, unclear direction of association and unadjusted confounding effect. Therefore, it is of great significance to adopt advanced method to comprehensively determine the causal associations between these lifestyle factors and SIS.

Mendelian randomization (MR) employs genetic variants (single nucleotide polymorphisms, SNPs) as instruments to infer the causal relationship between exposure and the disease of interest [17]. Underlying assumptions include the relevance of the genetic variant to the exposure, the independence of the genetic variant from confounding factors, and the exclusion restriction principle. Because genetic variants are randomly assorted at conception and cannot be modified by the onset and progression of the disease, MR studies can overcome residual confounding and reduce reverse causality [17]. Two-sample MR analysis is an extension of the MR method, allowing the relationship of exposure and outcome summary statistics from genome-wide association studies (GWASs) to increase the sample size to enhance statistical power by forming consortia [18]. Due to the compelling advantages of MR study, we hypothesized that using a two-sample MR design is able to reduce residual confounding and reverse causality, thus determining the causality between lifestyle factors and SIS. This study aimed to identify the risk factors of SIS though inferring the causality relationships between lifestyle factors and SIS by a MR study design, which may be useful for establishing the strategies of targeting lifestyle factors in the management of SIS patients.

## Methods

### Data source

Original GWAS is a type of genetic research used to identify genetic variations associated with a particular trait or disease, corresponding results were aggregated into GWAS summary statistics. This study is a two-sample MR approach based on summary-level GWAS summary statistics [18]. All these GWAS data were publicly available, including the UK Biobank (UKB) and FinnGen Study. Each GWAS in these studies has been approved by relevant ethical review committees. The UKB is a large-scale prospective cohort study that recruited 502,682 UK participants aged 40–69 from 2006 to 2010 in the UK, with ongoing follow-up, and included extensive health, lifestyle, and genetic data [19]. FinnGen study, a Finnish collaboration since 2017, aims to reach 500,000 Finnish participants with long-term follow-up, focusing on health records, genetic sequencing, and various -omics data. Through access to national health registries, the FinnGen cohorts provide summary statistics of different endpoints, linked with survival analyses and drug statistics. The FinnGen Study had no to very limited overlap in population with the UKB, which can be acceptable for two-sample MR analysis [20].

In this study, GWAS data of exposures were obtained from UKB. The Pan-UKB team collected these GWAS data for further research (https://pan.ukbb.broadinstitute.org/downloads/index.html). We considered a total of 11 modifiable lifestyle factors, including smoking initiation, alcoholic drinks per week, coffee consumption, moderate-to-vigorous physical activity, sedentary behavior, insomnia, sleep duration, short sleep duration, time spent using computers, weekly usage of mobile phone in the last 3 months, and job involving heavy manual or physical work. In this study, smoking initiation was selected since it had a significant impact on the duration and intensity of smoking behavior over time [21]. Two-sample MR analysis requires two non-overlapping datasets to avoid an inflated type I error [22]. In our MR study, we obtained the GWAS data of SIS from the FinnGen cohort. The GWAS data of SIS involved 5,139 patients with SIS (cases) and 167,641 controls without any history of musculoskeletal system disease (controls) (https://gwas.mrcieu.ac.uk/datasets/finn-b-M13_IMPINGEMENT/). The diagnosis of SIS was made based on the 10th edition International Classification of Diseases (ICD-10) code M75.4, where SIS is defined as compression of the rotator cuff tendons and subacromial bursa between the humeral head and structures that make up the coracoacromial arch and the humeral tuberosities [23]. The GWAS data was adjusted for age, sex, genotyping batch, and 10 principal components (PCs) [20].

### Selection of instrumental variable (IV)

To assess the causal association of lifestyle factors with SIS, we used a two-sample MR approach, assuming that: (i) SNPs selected as instruments were strongly associated with exposures; (ii) SNPs were independent of any confounder between the exposures and outcome; (iii) the SNPs affect the outcome only through the exposure, rather than via any other pathway. We selected robustly independent SNPs (r^2^ < 0.001, clump window of 10,000 kb) and genome-wide significant (*P* < 5.0 × 10^− 8^) from GWAS data of each lifestyle factor to guarantee assumption i. We removed the SNPs that were not present in the summary-level dataset of the outcome. Palindromic with intermediate allele frequencies were also excluded during the harmonization process. Detailed descriptions of selected SNPs along with the definition of the unit of each exposure were presented in Table 1. The phenotypic variance explained by selected SNPs ranged from 0.14% for sedentary behavior to 4.8% for smoking initiation. The mean F-statistic for each exposure was calculated using the previously specified approximation method [24], and the results showed the mean F-statistic varied from 23 for time spent using computers to 191 for short sleep duration. The main characteristic of selected SNPs for each exposure were listed in Supplementary Tables 1 to 11.

**Table 1:** Main characteristic of data sources of the instrumental variables used in the MR study

Risk factors	SNPs	Sample size	Ancestry	Unit	PubMed ID/GWAS ID
Smoking initiation	334	1,232,091	European	Ever smoked regularly compared with never smoked	30,643,251/NA
Alcoholic drinks per week	89	46,568	European	1 SD increase in log-transformed alcoholic drinks/week	30,643,251/NA
Coffee consumption	14	375,833	European	50% change in cups of coffee intake per day	31,046,077/NA
Moderate-to-vigorous physical activity	18	377,234	European	1 SD increase in MET min/week	29,899,525/ebi-a-GCST006097
Sedentary behavior	4	91,105	European	1 SD increase in sedentary time	30,531,941/NA
Insomnia	227	1,331,010	European	Not available for binary trait	30,804,565/NA
Sleep duration	70	446,118	European	h/day	30,846,698/NA
Short sleep duration	24	411,934	European	< 7 h vs. 7–8 h daily sleep duration	30,846,698/NA
Time spent using computers	76	360,895	European	1 SD increase in time spent using computers	NA/ukb-b-4522
Weekly usage of mobile phones in last 3 months	11	386,626	European	1 SD increase in time using mobile phones	NA/ukb-b-17,999
Job involves heavy manual or physical work	21	263,615	European	1 SD increase in frequency of heavy manual or physical work	NA/ukb-b-2002

SNP: single nucleotide polymorphism; GWAS: genome-wide association study; SD: standard deviation; NA: not available

### Statistical analysis

All the analyses were performed with R (version 4.1.3), TwoSampleMR (version 0.5.5) [25], Mendelian Randomization (version 0.5.0) [26], and MR-PRESSO package [27]. For the primary MR analyses, we adopted the inverse-variance weighted (IVW) method under the random-effect model. The IVW method combines the Wald ratio of each individual selected SNP using the meta-analytic approach to provide an estimated causal effect, where Wald ratio was the causal estimate of a SNP by dividing its gene-outcome association by its gene-exposure association. Meanwhile, power analyses for the was conducted for the 11 MR analyses based on binary outcome [28].

Even though the IVW method gives the most precise estimation of causal effects, it is sensitive to invalid instruments having pleiotropic effects [29]. Therefore, we planned and performed several sensitivity analyses including the weighted median method, the MR-Egger method, and the MR-PRESSO method, which relax some instrument assumptions. However, no study protocol was pre-registered, their results should only be explained to evaluate robustness of our findings. The weighted median method provides a constant estimate of causal effect given that more than half of the weight is derived from valid instruments [24]. The MR-Egger regression analysis was initially designed for meta-analysis, it allows horizontal pleiotropy by introducing the concept of Egger intercept, meanwhile, test on non-zero intercept from MR-Egger regression can be used to test pleiotropy [24]. If the intercept is significantly different from zero, it suggests that there is evidence of horizontal pleiotropy, which violates the assumption that the SNPs are valid instruments (assumption ii) and that there are no residual confounders after controlling for the exposure (assumption iii). The MR-PRESSO approach identifies outlying SNPs (MR-PRESSO global test) and provides an estimate of outlier-corrected causal association [27]. The P value for the MR-PRESSO distortion test was used to indicate whether the outlier-corrected estimate differs from the original one [27]. Heterogeneity among selected SNPs was tested using Cochrane’s Q-statistics, its P-value less than 0.10 indicted existence of heterogeneity [29].

The pre-specified significance level was set as 0.05. To account for multiple testing, we used a Bonferroni-corrected P value of 0.0045 (0.05 divided by 11 lifestyle factors). P value from MR analysis less than 0.0045 was considered as statistical significance. P value between 0.0045 and 0.05 was deemed as suggestive significant. The results of MR analyses were reported as odds ratio (OR) and its associated 95% confidence interval (95%CI). All the P values were two-sided.

## Results

**Fig. 1:**
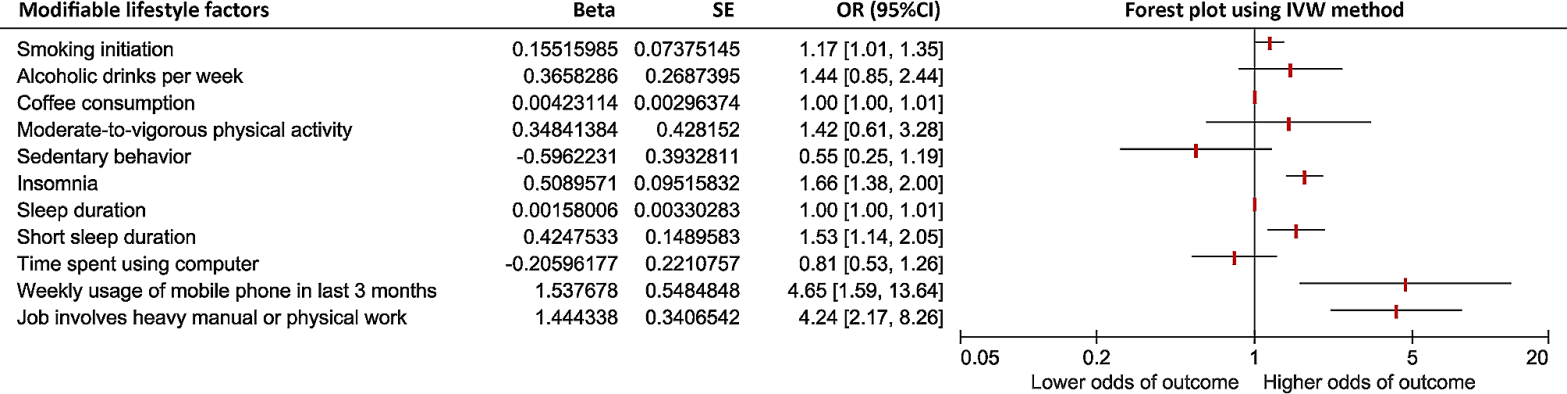
Forest plot of the associations between modifiable lifestyle factors (exposure) and subacromial impingement syndrome of shoulder (outcome); The odds of subacromial impingement syndrome of shoulder was compared between different levels of lifestyle factor

A significantly higher OR for SIS was found for the following four modifiable lifestyle factors in the MR analyses using the IVW method **(**Fig. 1**and** Table 2**)**: genetically predicted insomnia (OR: 1.66 95%CI 1.38, 2.00; *P* = 8.86 × 10^− 8^), short sleep duration (OR: 1.53 95%CI 1.14, 2.05: *P* = 0.0043), weekly usage of mobile phone in last 3 months (OR: 4.65, 95%CI 1.59, 13.64; *P* = 0.0051), and job involving heavy manual or physical work (OR: 4.24, 95%CI 2.17, 8.26; *P* = 0.000022). A suggestive causal association was found between smoking initiation and SIS (OR: 1.17, 95%CI 1.01, 1.35; *P* = 0.035). No significant associations were detected for genetically predicted alcoholic drinks per week, coffee consumption, moderate-to-vigorous physical activity, sedentary behavior, sleep duration, and time spent using computers. Using the IVW method, significant heterogeneity across selected SNPs was observed for smoking initiation, alcoholic drinks per week, insomnia, and weekly usage of mobile phones in the last three months.

**Table 2:** Causal effects of modifiable lifestyle factors on impingement syndrome of shoulder using MR analyses

Exposure	MR method	OR (95%CI)	Beta (SE)	P	P_− het_	P_− pleio_
Smoking initiation	MR Egger	0.91 (0.50, 1.67)	-0.089 (0.31)	0.77	0.0068	
	MR Egger intercept		0.0048 (0.0059)			0.41
	Weighted median	1.11 (0.90, 1.35)	0.10 (0.10)	0.33		
	IVW	1.17 (1.01, 1.35)	0.16 (0.074)	0.035	0.0069	
	MR-PRESSO distortion test			NA		
Alcoholic drinks per week	MR Egger	3.59 (1.00, 12.86)	1.28 (0.65)	0.052	0.0023	
	MR Egger intercept		-0.012 (0.0078)			0.13
	Weighted median	1.97 (0.96, 4.02)	0.68 (0.36)	0.065		
	IVW	1.44 (0.85, 2.44)	0.37 (0.27)	0.17	0.0015	
	MR-PRESSO distortion test			0.80		
Coffee consumption	MR Egger	1.01 (1.00, 1.02)	0.0094 (0.0057)	0.12	0.95	
	MR Egger intercept		-0.014 (0.014)			0.31
	Weighted median	1.01 (1.00, 1.01)	0.0061 (0.0038)	0.12		
	IVW	1.00 (1.00, 1.01)	0.0042 (0.0030)	0.15	0.93	
	MR-PRESSO distortion test			NA		
Moderate-to-vigorous physical activity	MR Egger	21.76 (0.20, 2428.16)	1.08 (2.41)	0.22	0.18	
	MR Egger intercept		-0.041 (0.035)			0.27
	Weighted median	1.04 (0.36, 2.95)	0.039 (0.53)	0.87		
	IVW	1.42 (0.61, 3.28)	0.35 (0.43)	0.42	0.16	
	MR-PRESSO distortion test			NA		
Sedentary behavior	MR Egger	3.85 (0.093, 159.16)	1.34 (1.90)	0.55	0.79	
	MR Egger intercept		-0.063 (0.060)			0.40
	Weighted median	0.54 (0.22, 1.35)	-0.61 (0.46)	0.20		
	IVW	0.55 (0.25, 1.19)	-0.60 (0.39)	0.13	0.67	
	MR-PRESSO distortion test			NA		
Insomnia	MR Egger	1.59 (0.76, 3.32)	0.46 (0.38)	0.22	0.0018	
	MR Egger intercept		0.00093 (0.0071)			0.90
	Weighted median	1.52 (1.16, 2.00)	0.42 (0.14)	0.0025		
	IVW	1.66 (1.38, 2.00)	0.51 (0.10)	8.86E-08	0.0021	
	MR-PRESSO distortion test			0.78		
Sleep duration	MR Egger	1.00 (0.97, 1.02)	-0.0043 (0.13)	0.74	0.087	
	MR Egger intercept		0.0059 (0.013)			0.64
	Weighted median	1.00 (0.99, 1.01)	-0.0062 (0.045)	0.56		
	IVW	1.00 (1.00, 1.01)	0.0016 (0.0033)	0.63	0.098	
	MR-PRESSO distortion test			NA		
Short sleep duration	MR Egger	1.59 (0.43, 5.90)	0.46 (0.67)	0.50	0.50	
	MR Egger intercept		-0.0013 (0.023)			0.95
	Weighted median	1.41 (0.92, 2.16)	0.34 (0.22)	0.12		
	IVW	1.53 (1.14, 2.05)	0.42 (0.15)	0.0043	0.56	
	MR-PRESSO distortion test			NA		
Time spent using computers	MR Egger	0.56 (0.049, 6.50)	-0.58 (1.25)	0.65	0.29	
	MR Egger intercept		0.0047 (0.016)			0.76
	Weighted median	1.08 (0.60, 1.95)	0.074 (0.30)	0.81		
	IVW	0.81 (0.53, 1.26)	-0.21 (0.22)	0.35	0.31	
	MR-PRESSO distortion test			NA		
Weekly usage of mobile phones in last 3 months	MR Egger	6.71 (0.0045, 9908.00)	1.90 (3.72)	0.62	0.0031	
	MR Egger intercept		-0.0075 (0.075)			0.92
	Weighted median	5.46 (1.88, 15.91)	1.70 (0.55)	0.00019		
	IVW	4.65 (1.59, 13.64)	1.54 (0.55)	0.0051	0.0055	
	MR-PRESSO distortion test			0.50		
Job involving heavy manual or physical work	MR Egger	0.12 (0.0031, 4.72)	-2.11 (1.87)	0.27	0.54	
	MR Egger intercept		0.058 (0.030)			0.068
	Weighted median	4.25 (1.70, 10.67)	1.44 (0.47)	0.0033		
	IVW	4.24 (2.17, 8.26)	1.44 (0.34)	0.000022	0.37	
	MR-PRESSO distortion test			NA		

MR: mendelian randomization; OR: odds ratio; 95%CI: 95% confidence interval; Beta was the estimated effect size; SE: standard error; P_− het_: P value for heterogeneity test; P_− pleio_: P value for pleiotropy test using MR-Egger regression analysis; IVW: inverse variance weighting; *P* < 0.05 was considered statistically significant

For all the analyses, the MR-Egger intercepts were centered around zero, indicating no potential directional pleiotropy **(**Table 2**)**. In the sensitivity analyses using the weighted median method, significant causal associations were also found for genetically predicted insomnia (OR: 1.52 95%CI 1.16, 2.00; *P* = 0.0025), weekly usage of mobile phone in the last 3 months (OR: 5.46 95%CI 1.88, 15.91; *P* = 0.00019), and job involving heavy manual or physical work (OR: 4.25 95%CI 1.70, 10.67; *P* = 0.0033) **(**Table 2**)**. Albeit null association for smoking initiation (OR: 1.11 95%CI 0.90, 1.35; *P* = 0.33) and short sleep duration (OR: 1.41 95%CI 0.92, 2.16; *P* = 0.12) using weighted median method **(**Table 2**)**, the direction of association was in line with that derived from IVW method. As significant heterogeneity was present in several analyses, we also performed the MR-PRESSO analyses to identify outlying SNPs with potential pleiotropy. The MR-PRESSO method identified outlying SNPs in the analyses for alcoholic drinks per week, insomnia, and weekly usage of mobile phones in the last 3 months, and the associations remained unchanged after the correction for outliers **(**Table 2**)**.

## Discussions

This study is the first comprehensive MR analysis that establishes a causal relationship between lifestyle factors and SIS risk. The present MR study systematically assessed the potential causal role of 11 modifiable lifestyle factors in SIS based on MR method. In our MR anayses, strict selection of SNP and large F statistics could reduce weak instrument bias and 3 assumptions were evaluated to guarantee the validity of findings. Our study revealed causal associations of genetically predicted insomnia, short sleep duration, weekly usage of mobile phone in the last 3 months, and job involving heavy manual or physical work with an increased risk for SIS. We also observed a suggestive association between smoking initiation and higher SIS risk. Sensitivity analyses with relaxed assumptions also supported our findings.

### Insomnia/short sleep duration/sleep duration and SIS

Previous cross-sectional studies have supported positive associations of insomnia and sleep duration with increased risk of SIS [30, 31], but this is not completely consistent with our MR findings. We observed a significant effect of insomnia and short sleep duration, rather than sleep duration, on the increased SIS risk. Since the causal relationship between these lifestyle factors and SIS has not been illustrated before, people tend to regard insomnia and short sleep duration as the symptoms after SIS development [32, 33], and easily overlook that these bad lifestyles may be risk factors for the occurrence of SIS. Some prospective and cross-sectional studies attempted to attribute shoulder discomfort to insomnia and short sleep duration [34–37] but were limited by modest sample size, reverse causality, and the inability to mitigate confounding effects completely. Inspired by this, we conducted the present MR study and found that genetically predicted insomnia and short sleep duration are causally associated with a higher SIS risk. The mechanisms through which insomnia/short sleep duration causes SIS are unclear. However, studies have shown that insomnia/short sleep duration may lead to increased rates of physical inactivity [38, 39] and obesity [40, 41], both of which were reported to be associated with an increased risk of shoulder discomfort [42]. This may be due to the increased production of adipokines in physically inactive obese individuals, which induce oxidative stress, inflammation, thrombosis, and endothelial dysfunction, contributing to peripheral vascular deficiencies [43, 44]. Oxidative stress may cause cell apoptosis, thereby leading to a degeneration of the tendon and predisposing it to rupture [45, 46]. A longitudinal study of 26,896 individuals linked sleep problems to a higher risk of chronic shoulder pain and found that physical exercise and maintaining normal body weight may reduce the adverse effects of mild sleep problems on the risk of chronic shoulder pain [47]. Nevertheless, the specific pathway from insomnia/short sleep duration to increased risk of SIS has not been fully elucidated and more work is warranted to further decipher more comprehensive underlying mechanisms.

### Weekly usage of mobile phone in last 3 months and SIS

We also found evidence of a strong association between weekly usage of mobile phones in the last 3 months and SIS risk. Observational studies have found that mobile phone use time is associated with an increased risk of shoulder discomfort [48, 49], but SIS was not specifically mentioned. Our work suggested individuals with higher weekly mobile phone usage in the last 3 months were more likely to develop SIS. Previous studies have emphasized the increased muscle activity in the neck-shoulder regions when individuals use mobile phones [50, 51], while such chronic muscle strain may have a significant and cumulative effect that increases the risk of SIS episodes. A cross-sectional study of 8,990 adolescents reported that physical activity might play a protective role in the increased risk of shoulder pain associated with screen-based activities [52]. Although our work suggested a causal relationship between weekly usage of mobile phones in the last 3 months and SIS, to the best of our knowledge, there is a lack of population-based epidemiological studies directly exploring the role of weekly usage of mobile phones in the last 3 months in the increased SIS risk. Therefore, more independent lines of evidence are needed to validate our MR results, and the latent mechanism of this causal effect also needs further investigation.

### Job involving heavy manual or physical work and SIS

Occupational exposures were associated with higher SIS risk. A meta-analysis of 17 studies found that forceful exertion in work (force requirements > 10% maximal voluntary contraction, lifting > 20 kg > 10 times/day, and high level of hand force > 1 h/day) was associated with the occurrence of SIS [53]. This was consistent with subsequent randomized controlled and observational studies demonstrating a generally positive association between jobs involving heavy manual physical work and the risk of surgery for SIS [10, 44, 54–57]. Our results were in line with previous studies and supported that those whose jobs involve heavy manual or physical work are more likely to suffer from SIS. Therefore, long-term exposure to jobs involving heavy manual or physical work should be advised to prevent the occurrence of SIS in advance, and workers with SIS should be advised to avoid or at least reduce harmful exposure to the shoulder.

### Other lifestyle factors and SIS

Studies on smoking initiation concerning SIS risk are scarce. A case-control study involving 111 SIS patients and 191 healthy volunteers evaluated the association between smoking initiation and the occurrence of SIS and found that current smokers had a 6.8 times higher risk of SIS compared with non-smokers [8]. Our results show a suggestive positive association between genetically predicted smoking initiation and an increased risk of SIS. Given these inconsistent results, and the fact that SIS data come from different populations, more research is needed to examine the association between smoking initiation and SIS risk. Associations of alcoholic drinks per week, coffee consumption, moderate-to-vigorous physical activity, sedentary behavior, and time spent using computers with SIS risk has not been investigated, and the results of the present MR study show that there is no causal association between these five lifestyle factors and SIS risk.

### Significance of the current study

Our current work identified certain causal effects of insomnia, short sleep duration, weekly usage of mobile phones in the last 3 months, and job involving heavy manual or physical work-related phenotypes in the issue of SIS. However, it should be noted that these causal estimates should not be interpreted as the expected effect of clinical interventions for risk factors but can be used to help us screen the populations at risk for SIS. Furthermore, the current causal inference using MR design strongly supports the subsequent conduct of relevant randomized controlled trials to further determine the impact of interventions on the development of SIS.

### Strengths and limitations

This MR study has several strengths and limitations. First, the major strength was that we used an MR design to reduce residual confounding and reverse causality, thereby improving the assessment of causal associations between the 11 lifestyle factors and SIS. Second, in addition to the traditional IVW methods, we adopted the weighted median method, MR-Egger method, and MR-PRESSO method as sensitivity analyses to ensure the consistency of causal estimates, which supported the robustness of causal inference in this study. Third, bias due to demographic stratification was minimized by confining all analyses within datasets of European ancestry. There are several limitations of the present study. First, this restriction on the European populations may limit the generalizability of our findings to other populations. As a result, the causal associations of insomnia, short sleep duration, weekly usage of mobile phone in the last 3 months, and job involving heavy manual or physical work with SIS risk in other populations remains unclear. Second, the current analysis focused solely on SIS (ICD-10 code M75.4) as the primary endpoint within the FinnGen cohort. While SIS may be interchangeably used with bursitis and rotator cuff disease, future studies could benefit from investigating the additional interchangeable endpoints, such as bursitis of shoulder (M75.5) and rotator cuff syndrome (M75.1), to provide a more comprehensive understanding and strengthen the generalizability of our findings. Third, our findings only report the effects of four lifestyle factors on the increased risk of SIS, but the underlying mechanisms warrant further investigation. Meanwhile, future studies may explore the inclusion of more comprehensive lifestyle factors, such as number of cigarettes per day. Finally, even though we have employed stringent SNP selection criteria, utilized robust MR methods and conducted sensitivity analyses, we must acknowledge that the validity of our findings was based on strict adherence to three key assumptions and gene-environment equivalence assumption [58]. Little studies can completely guarantee the fulfillment of all MR assumptions [59]. We have taken measures to reduce these limitations, but the risk of residual and the challenge of unobserved environmental confounding and pleiotropy effect might be not ruled out entirely, which limited the interpretation of our findings, future research with additional data on environmental exposures and their potential interactions with genetic variants would be valuable. Because of the use of summary-level data from UKB and FinnGen, we could not perform further gender or age stratification analysis.

## Conclusion

In conclusion, this MR study suggests that insomnia, short sleep duration, weekly usage of mobile phones in the last 3 months, and job involving heavy manual or physical work are the risk factors for SIS. Based on the causal evidences, this study can helpful to formulating feasible strategies for SIS screening and prevention, while also offering evidence-based recommendations for lifestyle modifications in SIS management. These recommendations may include interventions aimed at improving sleep quality and duration, reducing excessive mobile phone usage, and implementing ergonomic strategies in occupations involving heavy manual or physical work.

### Electronic supplementary material

Below is the link to the electronic supplementary material.

Supplementary Material 1

## Data Availability

Data are available in supplementary materials and online databases.
