# Supplementary material for: Lifestyle factors and subacromial impingement syndrome of the shoulder: potential associations in finnish participants
Source: BMC Musculoskelet Disord. 2024 Mar 19;25:220. doi: 10.1186/s12891-024-07345-w (PMC10949643; doi:10.1186/s12891-024-07345-w)
Supplement: Supplementary file 1 — Supplementary Material 1 [file 12891_2024_7345_MOESM1_ESM.docx]

**Supplementary Materials**

**Figure legend**

**Supplementary Table 1.** Main characteristics of SNPs to instrument smoking initiation.

**Supplementary Table 2.** Main characteristics of SNPs to instrument alcoholic drinks per week.

**Supplementary Table 3.** Main characteristics of SNPs to instrument coffee consumption.

**Supplementary Table 4.** Main characteristics of SNPs to instrument moderate-to-vigorous physical activity.

**Supplementary Table 5.** Main characteristics of SNPs to instrument sedentary behavior.

**Supplementary Table 6.** Main characteristics of SNPs to instrument insomnia.

**Supplementary Table 7.** Main characteristics of SNPs to instrument sleep duration.

**Supplementary Table 8.** Main characteristics of SNPs to instrument short sleep duration.

**Supplementary Table 9.** Main characteristics of SNPs to instrument time spent using computer.

**Supplementary Table 10.** Main characteristics of SNPs to instrument weekly usage of mobile phone in last 3 months.

**Supplementary Table 11.** Main characteristics of SNPs to instrument job involving heavy manual or physical work.

**Supplementary Table 1. Main characteristics of SNPs to instrument smoking initiation.**

| **SNP** | **Chr** | **Position** | **EA** | **OA** | **EAF** | **Beta** | **SE** | **P value** | **Sample size** | **R^2^** | **F** |
| --- | --- | --- | --- | --- | --- | --- | --- | --- | --- | --- | --- |
| rs1008078 | 1 | 91189731 | C | T | 0.402 | -0.0228169 | 0.002599 | 1.63E-18 | 1232091 | 0.0002503 | 308.48 |
| rs1022528 | 1 | 71490122 | G | A | 0.344 | -0.0174022 | 0.002682 | 8.48E-11 | 1232091 | 0.0001367 | 168.42 |
| rs10789369 | 1 | 73824909 | A | G | 0.615 | 0.02344838 | 0.002618 | 3.39E-19 | 1232091 | 0.0002604 | 320.88 |
| rs10873871 | 1 | 76689019 | A | G | 0.207 | -0.0174521 | 0.003145 | 2.82E-08 | 1232091 | 9.999E-05 | 123.21 |
| rs10914684 | 1 | 33795572 | G | A | 0.324 | 0.01580384 | 0.002722 | 6.32E-09 | 1232091 | 0.0001094 | 134.81 |
| rs11162019 | 1 | 87913176 | C | T | 0.363 | 0.01549472 | 0.00265 | 5.06E-09 | 1232091 | 0.000111 | 136.81 |
| rs11587399 | 1 | 50861071 | A | T | 0.221 | 0.01780456 | 0.003076 | 7.25E-09 | 1227673 | 0.0001091 | 134.01 |
| rs12022778 | 1 | 50603995 | A | C | 0.202 | -0.0268248 | 0.003179 | 3.18E-17 | 1227673 | 0.000232 | 284.87 |
| rs12130857 | 1 | 7791461 | G | A | 0.324968 | 0.01800314 | 0.00272 | 3.65E-11 | 1232091 | 0.0001422 | 175.22 |
| rs12563365 | 1 | 236872829 | G | A | 0.556 | -0.0165589 | 0.002564 | 1.05E-10 | 1232091 | 0.0001354 | 166.82 |
| rs12739243 | 1 | 210302043 | T | C | 0.221 | 0.02125184 | 0.003071 | 4.45E-12 | 1232091 | 0.0001555 | 191.63 |
| rs12740789 | 1 | 72752073 | G | A | 0.178 | 0.02849727 | 0.003331 | 1.18E-17 | 1232091 | 0.0002376 | 292.87 |
| rs12755632 | 1 | 41776623 | A | G | 0.316 | 0.01540514 | 0.00274 | 1.93E-08 | 1232091 | 0.0001026 | 126.41 |
| rs147052174 | 1 | 179783167 | G | T | 0.0171 | -0.0623094 | 0.009827 | 2.3E-10 | 1232091 | 0.0001305 | 160.82 |
| rs1514176 | 1 | 74991596 | G | A | 0.58 | 0.01930016 | 0.002581 | 7.67E-14 | 1232091 | 0.0001815 | 223.64 |
| rs1889571 | 1 | 32195819 | T | G | 0.131 | -0.0221798 | 0.003776 | 4.19E-09 | 1232091 | 0.000112 | 138.02 |
| rs1935571 | 1 | 96414335 | T | G | 0.48 | 0.01572038 | 0.00255 | 6.99E-10 | 1232091 | 0.0001234 | 152.02 |
| rs2901785 | 1 | 174104743 | G | A | 0.446 | 0.01730827 | 0.002563 | 1.47E-11 | 1232091 | 0.000148 | 182.43 |
| rs301807 | 1 | 8484823 | A | G | 0.57 | -0.0180144 | 0.002573 | 2.5E-12 | 1232091 | 0.0001591 | 196.03 |
| rs35656245 | 1 | 190957480 | G | A | 0.276 | -0.0159456 | 0.00285 | 2.23E-08 | 1232091 | 0.0001016 | 125.21 |
| rs3820277 | 1 | 18436657 | G | T | 0.526 | 0.01883692 | 0.002552 | 1.57E-13 | 1232091 | 0.0001769 | 218.04 |
| rs45444697 | 1 | 155034632 | C | G | 0.212 | -0.0196901 | 0.003117 | 2.72E-10 | 1232091 | 0.0001295 | 159.62 |
| rs4912332 | 1 | 58815243 | C | T | 0.491 | -0.0141209 | 0.002549 | 2.94E-08 | 1232091 | 9.967E-05 | 122.81 |
| rs876793 | 1 | 237852083 | T | C | 0.349262 | 0.0179245 | 0.002737 | 5.69E-11 | 1174994 | 0.000146 | 171.62 |
| rs925524 | 1 | 46496709 | A | G | 0.71 | -0.0155573 | 0.002808 | 2.94E-08 | 1232091 | 9.967E-05 | 122.81 |
| rs951740 | 1 | 44011737 | G | A | 0.625 | -0.0295409 | 0.002632 | 3.82E-29 | 1232091 | 0.0004091 | 504.21 |
| rs1004787 | 2 | 45159091 | G | A | 0.552 | -0.0284143 | 0.002562 | 1.11E-28 | 1232091 | 0.0003993 | 492.20 |
| rs1022376 | 2 | 22067213 | T | C | 0.515821 | 0.01474484 | 0.002611 | 1.66E-08 | 1174994 | 0.0001086 | 127.61 |
| rs10490159 | 2 | 51341259 | C | T | 0.394 | -0.0172365 | 0.002607 | 3.86E-11 | 1232091 | 0.0001419 | 174.82 |
| rs11678980 | 2 | 162101261 | G | A | 0.45 | -0.0176689 | 0.002561 | 5.19E-12 | 1232091 | 0.0001545 | 190.43 |
| rs11692435 | 2 | 98275354 | G | A | 0.0848 | -0.0250526 | 0.004582 | 4.47E-08 | 1227673 | 9.742E-05 | 119.61 |
| rs11889814 | 2 | 104432494 | A | C | 0.128 | 0.02102651 | 0.003814 | 3.44E-08 | 1232091 | 9.869E-05 | 121.61 |
| rs12474587 | 2 | 162802993 | G | T | 0.429 | -0.0242306 | 0.002574 | 4.83E-21 | 1232091 | 0.0002876 | 354.50 |
| rs12714017 | 2 | 80999398 | T | C | 0.511 | -0.0153965 | 0.00261 | 3.65E-09 | 1174994 | 0.0001185 | 139.22 |
| rs13007361 | 2 | 166250244 | G | A | 0.208 | -0.0175338 | 0.003139 | 2.29E-08 | 1232091 | 0.0001013 | 124.81 |
| rs13392222 | 2 | 100672408 | A | C | 0.139 | 0.02343755 | 0.003683 | 1.93E-10 | 1232091 | 0.0001315 | 162.02 |
| rs1445649 | 2 | 155682556 | T | C | 0.538 | -0.0205716 | 0.002556 | 8.48E-16 | 1232091 | 0.0002104 | 259.25 |
| rs16826827 | 2 | 147825689 | T | C | 0.124 | 0.02220689 | 0.003866 | 9.17E-09 | 1232091 | 0.0001071 | 132.01 |
| rs1722666 | 2 | 161816880 | C | T | 0.732 | -0.0160932 | 0.002877 | 2.17E-08 | 1232091 | 0.0001016 | 125.21 |
| rs17229285 | 2 | 199523122 | C | T | 0.505 | 0.01547956 | 0.002548 | 1.27E-09 | 1232091 | 0.0001198 | 147.62 |
| rs17616642 | 2 | 59022210 | A | G | 0.24687 | 0.01655729 | 0.002955 | 2.1E-08 | 1232091 | 0.0001019 | 125.61 |
| rs1863161 | 2 | 60139524 | G | A | 0.56094 | -0.0153394 | 0.002567 | 2.34E-09 | 1232091 | 0.0001159 | 142.82 |
| rs1901477 | 2 | 104126983 | A | G | 0.511 | -0.030437 | 0.00261 | 2.07E-31 | 1174994 | 0.000463 | 544.25 |
| rs2539706 | 2 | 59819545 | G | A | 0.529947 | -0.0162454 | 0.002553 | 1.95E-10 | 1232091 | 0.0001315 | 162.02 |
| rs2710634 | 2 | 32808804 | T | C | 0.521 | 0.01776143 | 0.00255 | 3.36E-12 | 1232091 | 0.0001575 | 194.03 |
| rs3115418 | 2 | 200936399 | T | C | 0.454 | 0.01422488 | 0.002559 | 2.79E-08 | 1232091 | 0.0001003 | 123.61 |
| rs34399632 | 2 | 137571174 | A | G | 0.232 | -0.0193504 | 0.003018 | 1.46E-10 | 1232091 | 0.0001334 | 164.42 |
| rs357304 | 2 | 164862639 | T | C | 0.727 | -0.0166757 | 0.00286 | 5.4E-09 | 1232091 | 0.0001104 | 136.01 |
| rs359247 | 2 | 60477052 | A | T | 0.638652 | -0.0220304 | 0.002652 | 9.89E-17 | 1232091 | 0.000224 | 276.06 |
| rs3811038 | 2 | 113240183 | T | C | 0.279 | -0.0191404 | 0.002841 | 1.58E-11 | 1232091 | 0.0001474 | 181.63 |
| rs4674916 | 2 | 225365635 | C | A | 0.327671 | 0.01802615 | 0.002714 | 3.06E-11 | 1232091 | 0.0001432 | 176.42 |
| rs4674993 | 2 | 226332033 | A | G | 0.2 | 0.02400534 | 0.003185 | 4.85E-14 | 1232091 | 0.0001844 | 227.24 |
| rs56208390 | 2 | 83247997 | A | G | 0.123 | -0.0215636 | 0.003879 | 2.68E-08 | 1232091 | 0.0001003 | 123.61 |
| rs61533748 | 2 | 22582968 | T | C | 0.384 | -0.0174357 | 0.00262 | 2.82E-11 | 1232091 | 0.0001438 | 177.23 |
| rs62106258 | 2 | 417167 | T | C | 0.047329 | 0.04549801 | 0.006 | 3.33E-14 | 1232091 | 0.0001867 | 230.04 |
| rs62137126 | 2 | 44250149 | A | G | 0.121094 | 0.02369109 | 0.003905 | 1.31E-09 | 1232091 | 0.0001195 | 147.22 |
| rs62180324 | 2 | 63416606 | G | A | 0.212 | 0.01951666 | 0.003117 | 3.91E-10 | 1232091 | 0.0001273 | 156.82 |
| rs62193862 | 2 | 202843875 | G | A | 0.0999 | -0.0238463 | 0.004249 | 1.99E-08 | 1232091 | 0.0001023 | 126.01 |
| rs6730325 | 2 | 59315828 | G | A | 0.609782 | 0.01463583 | 0.002612 | 2.1E-08 | 1232091 | 0.0001019 | 125.61 |
| rs6731872 | 2 | 624205 | T | G | 0.826 | -0.0315977 | 0.003361 | 5.35E-21 | 1232091 | 0.000287 | 353.70 |
| rs6750107 | 2 | 80748807 | G | A | 0.386875 | -0.0145651 | 0.002616 | 2.6E-08 | 1232091 | 0.0001006 | 124.01 |
| rs6750529 | 2 | 182027603 | C | T | 0.744 | -0.0199074 | 0.002919 | 9.26E-12 | 1232091 | 0.000151 | 186.03 |
| rs6756212 | 2 | 146140132 | C | T | 0.535 | 0.03388805 | 0.002554 | 3.49E-40 | 1232091 | 0.0005714 | 704.40 |
| rs72790288 | 2 | 29513404 | G | A | 0.0282 | 0.04553178 | 0.007696 | 3.28E-09 | 1232091 | 0.0001136 | 140.02 |
| rs74697736 | 2 | 145412271 | G | A | 0.287239 | -0.0222963 | 0.002816 | 2.43E-15 | 1232091 | 0.0002036 | 250.85 |
| rs75210106 | 2 | 113246436 | C | T | 0.176676 | 0.01865936 | 0.003341 | 2.33E-08 | 1232091 | 0.0001013 | 124.81 |
| rs7600835 | 2 | 172521827 | G | A | 0.342 | 0.01512158 | 0.002686 | 1.8E-08 | 1232091 | 0.0001029 | 126.81 |
| rs10446419 | 3 | 25725501 | A | G | 0.207 | 0.01956263 | 0.003145 | 5.05E-10 | 1232091 | 0.0001256 | 154.82 |
| rs10935779 | 3 | 149543102 | C | T | 0.415 | 0.01432718 | 0.002586 | 2.95E-08 | 1232091 | 9.967E-05 | 122.81 |
| rs11713899 | 3 | 2365026 | A | C | 0.171 | -0.0187188 | 0.003384 | 3.15E-08 | 1232091 | 9.934E-05 | 122.41 |
| rs1187820 | 3 | 173072584 | C | T | 0.439 | 0.01427116 | 0.002567 | 2.69E-08 | 1232091 | 0.0001003 | 123.61 |
| rs12053870 | 3 | 118302515 | T | G | 0.541511 | -0.0156164 | 0.002557 | 1.02E-09 | 1232091 | 0.0001211 | 149.22 |
| rs12633090 | 3 | 83241365 | G | C | 0.182 | 0.02301969 | 0.003302 | 3.16E-12 | 1232091 | 0.0001578 | 194.43 |
| rs13066050 | 3 | 81325861 | C | T | 0.208 | -0.0188343 | 0.003139 | 1.93E-09 | 1232091 | 0.0001169 | 144.02 |
| rs13319205 | 3 | 47800216 | T | A | 0.29 | -0.0165398 | 0.002808 | 3.77E-09 | 1232091 | 0.0001127 | 138.82 |
| rs1449012 | 3 | 159048333 | C | T | 0.463 | 0.01537341 | 0.002555 | 1.77E-09 | 1232091 | 0.0001175 | 144.82 |
| rs1549979 | 3 | 85460131 | C | T | 0.615 | 0.02452231 | 0.002623 | 8.8E-21 | 1227673 | 0.0002848 | 349.70 |
| rs16828799 | 3 | 173353739 | G | T | 0.156 | -0.0197692 | 0.003511 | 1.83E-08 | 1232091 | 0.0001029 | 126.81 |
| rs1714521 | 3 | 158284861 | A | C | 0.411 | 0.01629531 | 0.002589 | 3.07E-10 | 1232091 | 0.0001286 | 158.42 |
| rs1910236 | 3 | 59434420 | G | A | 0.469 | -0.0146439 | 0.002553 | 9.91E-09 | 1232091 | 0.0001068 | 131.61 |
| rs2196356 | 3 | 70890288 | G | C | 0.288886 | 0.01877276 | 0.002811 | 2.45E-11 | 1232091 | 0.0001448 | 178.43 |
| rs221988 | 3 | 64234307 | A | C | 0.384 | 0.01486502 | 0.00262 | 1.43E-08 | 1232091 | 0.0001045 | 128.81 |
| rs2276825 | 3 | 52886605 | T | C | 0.245 | -0.0188756 | 0.002962 | 1.89E-10 | 1232091 | 0.0001318 | 162.42 |
| rs2279829 | 3 | 147106319 | C | T | 0.216 | 0.01737657 | 0.003096 | 2.05E-08 | 1232091 | 0.0001023 | 126.01 |
| rs2306866 | 3 | 53766212 | A | T | 0.614 | 0.0166755 | 0.002617 | 1.89E-10 | 1232091 | 0.0001318 | 162.42 |
| rs2319545 | 3 | 147719648 | C | A | 0.149099 | -0.0232366 | 0.003577 | 8.3E-11 | 1232091 | 0.000137 | 168.82 |
| rs2734390 | 3 | 60459291 | A | G | 0.372 | -0.0147709 | 0.002636 | 2.09E-08 | 1232091 | 0.0001019 | 125.61 |
| rs3172494 | 3 | 48731487 | G | T | 0.115 | 0.02912668 | 0.004001 | 3.4E-13 | 1227673 | 0.0001727 | 212.04 |
| rs4543050 | 3 | 74954560 | A | T | 0.816 | -0.0222035 | 0.003288 | 1.45E-11 | 1232091 | 0.000148 | 182.43 |
| rs57153235 | 3 | 85902536 | T | G | 0.318 | 0.01937996 | 0.002741 | 1.56E-12 | 1227673 | 0.0001629 | 200.03 |
| rs62246017 | 3 | 71483084 | G | A | 0.322639 | 0.01616949 | 0.002725 | 3.03E-09 | 1232091 | 0.0001143 | 140.82 |
| rs6437769 | 3 | 107997514 | C | T | 0.581 | -0.0142141 | 0.002582 | 3.74E-08 | 1232091 | 9.837E-05 | 121.21 |
| rs6438436 | 3 | 117822149 | C | T | 0.816 | -0.024737 | 0.003288 | 5.33E-14 | 1232091 | 0.0001838 | 226.44 |
| rs6782116 | 3 | 77176032 | C | T | 0.415 | 0.01465022 | 0.002586 | 1.46E-08 | 1232091 | 0.0001042 | 128.41 |
| rs73831818 | 3 | 55988394 | A | G | 0.057 | -0.0320435 | 0.005495 | 5.46E-09 | 1232091 | 0.0001104 | 136.01 |
| rs748832 | 3 | 16851202 | A | G | 0.371 | -0.0172142 | 0.002637 | 6.6E-11 | 1232091 | 0.0001383 | 170.42 |
| rs7631379 | 3 | 181409057 | T | C | 0.206 | -0.0208014 | 0.00315 | 3.94E-11 | 1232091 | 0.0001415 | 174.42 |
| rs7640107 | 3 | 59966156 | C | T | 0.430789 | 0.01418605 | 0.002573 | 3.46E-08 | 1232091 | 9.869E-05 | 121.61 |
| rs9288999 | 3 | 114147927 | G | A | 0.735 | -0.0174411 | 0.002887 | 1.5E-09 | 1232091 | 0.0001185 | 146.02 |
| rs963354 | 3 | 157393770 | C | A | 0.687 | -0.0150489 | 0.002748 | 4.21E-08 | 1232091 | 9.74E-05 | 120.01 |
| rs9826984 | 3 | 131945722 | G | A | 0.542 | 0.01405286 | 0.002557 | 3.87E-08 | 1232091 | 9.804E-05 | 120.81 |
| rs9841807 | 3 | 175718927 | C | T | 0.273 | -0.0162535 | 0.00286 | 1.35E-08 | 1232091 | 0.0001049 | 129.21 |
| rs9850597 | 3 | 161761866 | G | A | 0.816 | 0.01857099 | 0.003288 | 1.65E-08 | 1232091 | 0.0001036 | 127.61 |
| rs1116690 | 4 | 143510148 | A | G | 0.742 | -0.0162912 | 0.002912 | 2.16E-08 | 1232091 | 0.0001016 | 125.21 |
| rs112725451 | 4 | 68017710 | C | T | 0.169 | -0.026092 | 0.0034 | 1.65E-14 | 1232091 | 0.0001912 | 235.64 |
| rs12642744 | 4 | 28027176 | G | T | 0.744 | 0.01659075 | 0.002989 | 2.82E-08 | 1174994 | 0.0001049 | 123.21 |
| rs13109980 | 4 | 140886963 | G | A | 0.326 | 0.02218155 | 0.002718 | 3.37E-16 | 1232091 | 0.0002162 | 266.46 |
| rs13110073 | 4 | 147797913 | T | C | 0.395 | 0.02464258 | 0.002606 | 3.24E-21 | 1232091 | 0.0002902 | 357.70 |
| rs1389171 | 4 | 28822284 | T | A | 0.241 | 0.01747203 | 0.002979 | 4.45E-09 | 1232091 | 0.0001117 | 137.62 |
| rs1435479 | 4 | 94550450 | G | T | 0.28748 | -0.0163905 | 0.002815 | 5.68E-09 | 1232091 | 0.0001101 | 135.61 |
| rs28717373 | 4 | 147985231 | C | T | 0.356165 | 0.0164657 | 0.002661 | 6.16E-10 | 1232091 | 0.0001243 | 153.22 |
| rs3934797 | 4 | 112467612 | G | A | 0.182 | 0.02129747 | 0.003302 | 1.12E-10 | 1232091 | 0.0001351 | 166.42 |
| rs58400863 | 4 | 31184484 | G | A | 0.347 | 0.02017188 | 0.002677 | 4.89E-14 | 1232091 | 0.0001844 | 227.24 |
| rs59537158 | 4 | 28246049 | C | T | 0.214 | -0.0224875 | 0.003107 | 4.62E-13 | 1232091 | 0.0001701 | 209.64 |
| rs62340589 | 4 | 176875795 | G | C | 0.201 | -0.0174134 | 0.003179 | 4.31E-08 | 1232091 | 9.74E-05 | 120.01 |
| rs71602617 | 4 | 136406155 | C | T | 0.216 | 0.01776549 | 0.00317 | 2.1E-08 | 1174994 | 0.0001069 | 125.61 |
| rs7657022 | 4 | 35501032 | A | G | 0.489 | -0.0182908 | 0.002549 | 7.34E-13 | 1232091 | 0.0001672 | 206.03 |
| rs7696257 | 4 | 137474783 | G | A | 0.366 | -0.0153313 | 0.002645 | 6.78E-09 | 1232091 | 0.0001091 | 134.41 |
| rs10060196 | 5 | 106455988 | C | A | 0.580606 | -0.0183116 | 0.002582 | 1.29E-12 | 1232091 | 0.0001633 | 201.23 |
| rs10805858 | 5 | 88873832 | A | T | 0.335286 | -0.0181241 | 0.002699 | 1.88E-11 | 1232091 | 0.0001464 | 180.43 |
| rs1173461 | 5 | 157707571 | C | T | 0.327 | -0.0166092 | 0.002716 | 9.51E-10 | 1232091 | 0.0001214 | 149.62 |
| rs11956866 | 5 | 161018271 | T | G | 0.567 | 0.01483817 | 0.002571 | 7.82E-09 | 1232091 | 0.0001081 | 133.21 |
| rs12517438 | 5 | 30842054 | T | G | 0.538 | -0.0153545 | 0.002556 | 1.89E-09 | 1232091 | 0.0001172 | 144.42 |
| rs1385108 | 5 | 154839646 | C | T | 0.239 | -0.0187045 | 0.002987 | 3.84E-10 | 1232091 | 0.0001273 | 156.82 |
| rs17165769 | 5 | 107365642 | A | G | 0.394872 | -0.0159396 | 0.002606 | 9.56E-10 | 1232091 | 0.0001214 | 149.62 |
| rs181508347 | 5 | 91366274 | T | G | 0.00965 | -0.0810758 | 0.013033 | 4.95E-10 | 1232091 | 0.0001256 | 154.82 |
| rs2028269 | 5 | 79308315 | G | A | 0.399 | -0.0161645 | 0.002602 | 5.19E-10 | 1232091 | 0.0001253 | 154.42 |
| rs2173019 | 5 | 167614971 | T | A | 0.177 | -0.028207 | 0.003338 | 2.98E-17 | 1232091 | 0.0002318 | 285.67 |
| rs329124 | 5 | 133865452 | A | G | 0.428 | 0.01638707 | 0.002575 | 1.96E-10 | 1232091 | 0.0001315 | 162.02 |
| rs35375873 | 5 | 43190647 | G | C | 0.11 | 0.02701025 | 0.004072 | 3.29E-11 | 1232091 | 0.0001428 | 176.02 |
| rs359431 | 5 | 173288534 | C | T | 0.56 | 0.01419822 | 0.002567 | 3.16E-08 | 1232091 | 9.934E-05 | 122.41 |
| rs3843905 | 5 | 165427280 | C | T | 0.403 | 0.01514584 | 0.002597 | 5.41E-09 | 1232091 | 0.0001104 | 136.01 |
| rs3909281 | 5 | 165096435 | T | G | 0.536 | -0.0210672 | 0.002555 | 1.62E-16 | 1232091 | 0.0002208 | 272.06 |
| rs4044321 | 5 | 166989513 | A | G | 0.644 | 0.02264097 | 0.002661 | 1.75E-17 | 1232091 | 0.000235 | 289.67 |
| rs42417 | 5 | 94198290 | C | T | 0.691 | -0.0169296 | 0.002757 | 8.27E-10 | 1232091 | 0.0001224 | 150.82 |
| rs6874731 | 5 | 80263865 | T | G | 0.484 | -0.0153179 | 0.002549 | 1.83E-09 | 1232091 | 0.0001172 | 144.42 |
| rs6890961 | 5 | 166778503 | C | T | 0.624 | 0.01931087 | 0.00263 | 2.13E-13 | 1232091 | 0.000175 | 215.64 |
| rs71592686 | 5 | 60121271 | T | C | 0.274 | -0.0207374 | 0.002857 | 3.85E-13 | 1232091 | 0.0001711 | 210.84 |
| rs72780746 | 5 | 103929588 | T | C | 0.173 | 0.02576296 | 0.003368 | 2.05E-14 | 1232091 | 0.0001899 | 234.04 |
| rs72789626 | 5 | 106825618 | T | A | 0.136 | 0.02564311 | 0.003717 | 5.13E-12 | 1232091 | 0.0001545 | 190.43 |
| rs79476395 | 5 | 166063680 | A | G | 0.0726 | -0.0333743 | 0.00491 | 1.04E-11 | 1232091 | 0.00015 | 184.83 |
| rs1059490 | 6 | 26171250 | T | C | 0.367 | 0.01859355 | 0.002648 | 2.16E-12 | 1227673 | 0.0001606 | 197.23 |
| rs10698713 | 6 | 158882320 | G | A | 0.0544 | 0.03351702 | 0.005617 | 2.38E-09 | 1232091 | 0.0001156 | 142.42 |
| rs10945141 | 6 | 69470709 | G | A | 0.263 | -0.0181417 | 0.002894 | 3.59E-10 | 1232091 | 0.0001276 | 157.22 |
| rs1150668 | 6 | 28129789 | T | G | 0.419 | 0.0185103 | 0.002587 | 8.54E-13 | 1227673 | 0.0001668 | 204.83 |
| rs118202 | 6 | 111658371 | G | T | 0.812 | 0.03674841 | 0.003261 | 1.9E-29 | 1232091 | 0.0004123 | 508.21 |
| rs12195240 | 6 | 98636905 | G | A | 0.285 | -0.0249108 | 0.002822 | 1.08E-18 | 1232091 | 0.0002529 | 311.68 |
| rs12530388 | 6 | 101329173 | A | C | 0.511 | 0.01836168 | 0.002549 | 5.83E-13 | 1232091 | 0.0001685 | 207.63 |
| rs160631 | 6 | 52895230 | T | G | 0.731 | 0.01726285 | 0.002873 | 1.87E-09 | 1232091 | 0.0001172 | 144.42 |
| rs1632941 | 6 | 29796685 | T | C | 0.46 | 0.0158074 | 0.002561 | 6.67E-10 | 1227673 | 0.0001241 | 152.42 |
| rs1737329 | 6 | 163807748 | C | G | 0.742 | -0.0170292 | 0.002912 | 5.08E-09 | 1232091 | 0.000111 | 136.81 |
| rs3218116 | 6 | 41901763 | C | T | 0.256 | 0.01984304 | 0.002919 | 1.05E-11 | 1232091 | 0.00015 | 184.83 |
| rs3800227 | 6 | 108994161 | A | G | 0.742 | -0.017178 | 0.002912 | 3.64E-09 | 1232091 | 0.000113 | 139.22 |
| rs619087 | 6 | 94175279 | A | G | 0.422 | -0.0142703 | 0.00258 | 3.1E-08 | 1232091 | 9.934E-05 | 122.41 |
| rs6568832 | 6 | 97702876 | G | A | 0.753851 | -0.018869 | 0.002958 | 1.74E-10 | 1232091 | 0.0001321 | 162.82 |
| rs6936160 | 6 | 100347745 | C | T | 0.698 | -0.0201068 | 0.002775 | 4.2E-13 | 1232091 | 0.0001704 | 210.04 |
| rs73008357 | 6 | 156431856 | A | C | 0.121 | 0.02230952 | 0.004 | 2.44E-08 | 1174994 | 0.0001059 | 124.41 |
| rs7743165 | 6 | 67521222 | T | G | 0.495 | -0.0192559 | 0.002548 | 4.15E-14 | 1232091 | 0.0001854 | 228.44 |
| rs9331343 | 6 | 157738258 | T | C | 0.568 | 0.01413452 | 0.002572 | 3.9E-08 | 1232091 | 9.804E-05 | 120.81 |
| rs10233018 | 7 | 117523709 | A | G | 0.516 | -0.0246124 | 0.002549 | 4.77E-22 | 1232091 | 0.0003026 | 372.91 |
| rs10272990 | 7 | 1703675 | T | C | 0.327622 | 0.02092154 | 0.002715 | 1.27E-14 | 1232091 | 0.0001928 | 237.65 |
| rs10279261 | 7 | 133589846 | G | A | 0.618 | 0.01887264 | 0.002622 | 6.05E-13 | 1232091 | 0.0001682 | 207.23 |
| rs1030015 | 7 | 78139581 | G | T | 0.519564 | -0.0142896 | 0.00255 | 2.15E-08 | 1232091 | 0.0001019 | 125.61 |
| rs10953957 | 7 | 121954709 | G | A | 0.386 | -0.0144058 | 0.002617 | 3.66E-08 | 1232091 | 9.837E-05 | 121.21 |
| rs112913817 | 7 | 115077394 | A | G | 0.011318 | -0.0780558 | 0.012044 | 9.28E-11 | 1232091 | 0.0001364 | 168.02 |
| rs11766326 | 7 | 111100585 | T | C | 0.506 | 0.01754396 | 0.00261 | 1.79E-11 | 1174994 | 0.0001539 | 180.83 |
| rs13437771 | 7 | 99071478 | A | G | 0.155 | 0.02710983 | 0.00352 | 1.39E-14 | 1232091 | 0.0001925 | 237.25 |
| rs1561112 | 7 | 133840652 | T | C | 0.412815 | 0.0152438 | 0.002588 | 3.84E-09 | 1232091 | 0.0001127 | 138.82 |
| rs1799068 | 7 | 97707069 | G | T | 0.379 | -0.0166096 | 0.002626 | 2.59E-10 | 1232091 | 0.0001299 | 160.02 |
| rs4727189 | 7 | 88442568 | T | C | 0.344 | -0.0148604 | 0.002682 | 3E-08 | 1232091 | 9.967E-05 | 122.81 |
| rs6968380 | 7 | 114940159 | G | A | 0.681 | 0.02341924 | 0.002734 | 1.05E-17 | 1232091 | 0.0002383 | 293.67 |
| rs76841737 | 7 | 91281409 | C | G | 0.103 | 0.02314881 | 0.004192 | 3.26E-08 | 1232091 | 9.902E-05 | 122.01 |
| rs77283305 | 7 | 132593831 | G | A | 0.305819 | 0.01519597 | 0.002765 | 3.91E-08 | 1232091 | 9.804E-05 | 120.81 |
| rs7802996 | 7 | 77771983 | C | T | 0.166 | 0.02088469 | 0.003424 | 1.06E-09 | 1232091 | 0.0001208 | 148.82 |
| rs7809303 | 7 | 69484366 | G | A | 0.325 | 0.02141884 | 0.00272 | 3.48E-15 | 1232091 | 0.0002013 | 248.05 |
| rs79631993 | 7 | 69432311 | A | C | 0.216281 | 0.01703435 | 0.003095 | 3.67E-08 | 1232091 | 9.837E-05 | 121.21 |
| rs11780471 | 8 | 27344719 | G | A | 0.063121 | 0.03867793 | 0.005239 | 1.57E-13 | 1232091 | 0.0001769 | 218.04 |
| rs11783093 | 8 | 27425349 | C | T | 0.158 | 0.04712428 | 0.003493 | 2.07E-41 | 1232091 | 0.0005909 | 728.43 |
| rs13261666 | 8 | 59814666 | G | T | 0.517 | 0.01999458 | 0.00255 | 4.36E-15 | 1232091 | 0.0001997 | 246.05 |
| rs1565735 | 8 | 27426077 | T | A | 0.204463 | 0.01916373 | 0.003159 | 1.33E-09 | 1232091 | 0.0001195 | 147.22 |
| rs2063976 | 8 | 91096366 | C | T | 0.664955 | 0.02018142 | 0.002699 | 7.45E-14 | 1232091 | 0.0001815 | 223.64 |
| rs290601 | 8 | 115374642 | C | T | 0.274 | -0.0163102 | 0.002857 | 1.14E-08 | 1232091 | 0.0001058 | 130.41 |
| rs2952251 | 8 | 10143164 | A | G | 0.74437 | -0.0164125 | 0.002997 | 4.24E-08 | 1170576 | 0.0001025 | 120.01 |
| rs6986430 | 8 | 93048104 | T | C | 0.222377 | 0.02433766 | 0.003064 | 1.99E-15 | 1232091 | 0.0002049 | 252.45 |
| rs6993429 | 8 | 92733282 | C | A | 0.453 | 0.01905047 | 0.002559 | 9.87E-14 | 1232091 | 0.0001799 | 221.64 |
| rs7836565 | 8 | 52569449 | C | T | 0.718 | 0.01550842 | 0.002831 | 4.36E-08 | 1232091 | 9.74E-05 | 120.01 |
| rs9987376 | 8 | 93190014 | T | G | 0.574251 | 0.02046824 | 0.002577 | 2.01E-15 | 1232091 | 0.0002049 | 252.45 |
| rs10858334 | 9 | 137989785 | C | G | 0.14 | -0.022871 | 0.00376 | 1.18E-09 | 1174994 | 0.000126 | 148.02 |
| rs10966092 | 9 | 23831658 | T | C | 0.267 | 0.02048619 | 0.00288 | 1.12E-12 | 1232091 | 0.0001643 | 202.43 |
| rs11791671 | 9 | 3398679 | C | T | 0.067315 | -0.0278504 | 0.005085 | 4.24E-08 | 1232091 | 9.74E-05 | 120.01 |
| rs1759433 | 9 | 128073097 | G | A | 0.48 | -0.0153647 | 0.00255 | 1.69E-09 | 1232091 | 0.0001178 | 145.22 |
| rs1927901 | 9 | 120519111 | T | C | 0.553 | 0.01417549 | 0.002563 | 3.1E-08 | 1232091 | 9.934E-05 | 122.41 |
| rs1930371 | 9 | 81444104 | C | T | 0.241 | 0.01724196 | 0.002979 | 7.09E-09 | 1232091 | 0.0001088 | 134.01 |
| rs2378662 | 9 | 86707289 | G | A | 0.541 | -0.0152121 | 0.002557 | 2.67E-09 | 1232091 | 0.0001149 | 141.62 |
| rs34553878 | 9 | 134334588 | A | G | 0.111 | -0.0246707 | 0.004056 | 1.17E-09 | 1232091 | 0.0001201 | 148.02 |
| rs3847244 | 9 | 3025368 | C | T | 0.47 | -0.0186717 | 0.002553 | 2.6E-13 | 1232091 | 0.0001737 | 214.04 |
| rs4837631 | 9 | 122061948 | C | T | 0.446 | 0.01535743 | 0.002563 | 2.03E-09 | 1232091 | 0.0001165 | 143.62 |
| rs4877285 | 9 | 81354129 | G | A | 0.668249 | 0.01813182 | 0.002706 | 2.1E-11 | 1232091 | 0.0001458 | 179.63 |
| rs7024924 | 9 | 8282399 | T | C | 0.174 | -0.0188918 | 0.003361 | 1.9E-08 | 1232091 | 0.0001026 | 126.41 |
| rs7026534 | 9 | 134907263 | T | G | 0.703821 | 0.01660302 | 0.002791 | 2.68E-09 | 1232091 | 0.0001149 | 141.62 |
| rs7867822 | 9 | 20676454 | A | G | 0.673 | 0.01509703 | 0.002716 | 2.76E-08 | 1232091 | 0.0001003 | 123.61 |
| rs10885480 | 10 | 115378364 | T | C | 0.284 | 0.0186775 | 0.002825 | 3.83E-11 | 1232091 | 0.0001419 | 174.82 |
| rs10905461 | 10 | 8803551 | T | C | 0.748 | 0.01639154 | 0.002935 | 2.36E-08 | 1232091 | 0.0001013 | 124.81 |
| rs11191269 | 10 | 104120522 | C | G | 0.193294 | -0.0176426 | 0.003226 | 4.61E-08 | 1232091 | 9.707E-05 | 119.61 |
| rs11192347 | 10 | 106929313 | G | A | 0.104 | 0.02645001 | 0.004274 | 6.15E-10 | 1174994 | 0.0001304 | 153.22 |
| rs11258417 | 10 | 13533053 | C | T | 0.391 | 0.01451362 | 0.002611 | 2.71E-08 | 1232091 | 0.0001003 | 123.61 |
| rs11594623 | 10 | 103960351 | T | C | 0.234241 | -0.0274396 | 0.003008 | 7.45E-20 | 1232091 | 0.0002701 | 332.89 |
| rs1291821 | 10 | 11133823 | A | G | 0.534 | -0.014493 | 0.002554 | 1.39E-08 | 1232091 | 0.0001045 | 128.81 |
| rs1733760 | 10 | 56698174 | T | C | 0.51 | -0.0147734 | 0.002549 | 6.7E-09 | 1232091 | 0.0001091 | 134.41 |
| rs2796793 | 10 | 36634124 | G | A | 0.452 | -0.0144814 | 0.00256 | 1.55E-08 | 1232091 | 0.0001039 | 128.01 |
| rs34970111 | 10 | 106078937 | C | T | 0.458 | 0.01455572 | 0.002557 | 1.28E-08 | 1232091 | 0.0001052 | 129.61 |
| rs4752018 | 10 | 118678712 | C | A | 0.231 | -0.0188538 | 0.003023 | 4.42E-10 | 1232091 | 0.0001263 | 155.62 |
| rs7072776 | 10 | 22032942 | A | G | 0.712 | 0.02197467 | 0.002814 | 5.66E-15 | 1232091 | 0.000198 | 244.05 |
| rs7901883 | 10 | 103186838 | G | A | 0.230322 | 0.01925745 | 0.003026 | 1.98E-10 | 1232091 | 0.0001315 | 162.02 |
| rs9423279 | 10 | 125680419 | C | G | 0.645 | 0.01858078 | 0.002663 | 3.06E-12 | 1232091 | 0.0001581 | 194.83 |
| rs9787523 | 10 | 106460460 | T | C | 0.418 | 0.01562732 | 0.002583 | 1.42E-09 | 1232091 | 0.0001188 | 146.42 |
| rs1106363 | 11 | 131966264 | C | T | 0.344579 | -0.0173746 | 0.002681 | 9.2E-11 | 1232091 | 0.0001364 | 168.02 |
| rs1381775 | 11 | 42442826 | T | C | 0.712 | 0.01561467 | 0.002814 | 2.79E-08 | 1232091 | 9.999E-05 | 123.21 |
| rs1713676 | 11 | 113660576 | A | G | 0.522512 | 0.01672625 | 0.002551 | 5.38E-11 | 1232091 | 0.0001396 | 172.02 |
| rs1834306 | 11 | 122023187 | A | G | 0.579399 | 0.01448521 | 0.002581 | 1.96E-08 | 1232091 | 0.0001023 | 126.01 |
| rs1944689 | 11 | 121634334 | G | T | 0.785911 | -0.01768 | 0.003106 | 1.27E-08 | 1232091 | 0.0001052 | 129.61 |
| rs2010921 | 11 | 132098205 | G | A | 0.311 | -0.0174291 | 0.002752 | 2.47E-10 | 1232091 | 0.0001302 | 160.42 |
| rs2155646 | 11 | 112912811 | T | C | 0.4 | -0.0377772 | 0.002601 | 9.44E-48 | 1232091 | 0.000685 | 844.58 |
| rs238896 | 11 | 113994505 | G | A | 0.49 | 0.01686738 | 0.002549 | 3.65E-11 | 1232091 | 0.0001422 | 175.22 |
| rs2939756 | 11 | 41436297 | G | A | 0.48 | 0.01569968 | 0.00255 | 7.45E-10 | 1232091 | 0.000123 | 151.62 |
| rs2959084 | 11 | 46078656 | G | A | 0.704674 | -0.0170799 | 0.002793 | 9.82E-10 | 1232091 | 0.0001214 | 149.62 |
| rs3740977 | 11 | 46393574 | T | C | 0.167 | -0.019474 | 0.003416 | 1.17E-08 | 1232091 | 0.0001055 | 130.01 |
| rs4275621 | 11 | 28652996 | A | G | 0.382 | 0.0213674 | 0.002622 | 3.76E-16 | 1232091 | 0.0002156 | 265.66 |
| rs540860 | 11 | 121530888 | A | G | 0.543 | -0.0176086 | 0.002558 | 5.75E-12 | 1232091 | 0.0001539 | 189.63 |
| rs586699 | 11 | 92289734 | G | A | 0.543 | 0.01480328 | 0.002558 | 7.29E-09 | 1232091 | 0.0001088 | 134.01 |
| rs61884449 | 11 | 64485193 | C | T | 0.149183 | -0.0199753 | 0.003576 | 2.32E-08 | 1232091 | 0.0001013 | 124.81 |
| rs61886926 | 11 | 64133552 | C | T | 0.384 | 0.01794007 | 0.00262 | 7.3E-12 | 1232091 | 0.0001523 | 187.63 |
| rs62618693 | 11 | 32956492 | C | T | 0.0428 | 0.03527242 | 0.006295 | 2.09E-08 | 1232091 | 0.0001019 | 125.61 |
| rs6265 | 11 | 27679916 | C | T | 0.188 | 0.02927549 | 0.003261 | 2.81E-19 | 1232091 | 0.0002617 | 322.48 |
| rs644740 | 11 | 65561468 | C | T | 0.457 | 0.01407852 | 0.002558 | 3.67E-08 | 1232091 | 9.837E-05 | 121.21 |
| rs76460663 | 11 | 111979741 | C | G | 0.041056 | 0.04234999 | 0.006421 | 4.15E-11 | 1232091 | 0.0001412 | 174.02 |
| rs7929518 | 11 | 85980958 | A | G | 0.773 | -0.0192363 | 0.003042 | 2.55E-10 | 1232091 | 0.0001299 | 160.02 |
| rs7943721 | 11 | 73309393 | G | A | 0.829 | 0.02121361 | 0.003384 | 3.58E-10 | 1232091 | 0.0001276 | 157.22 |
| rs11057005 | 12 | 16748721 | A | G | 0.441 | 0.0157139 | 0.002566 | 9.12E-10 | 1232091 | 0.0001217 | 150.02 |
| rs1109480 | 12 | 121083279 | G | A | 0.384 | 0.0166917 | 0.00262 | 1.84E-10 | 1232091 | 0.0001318 | 162.42 |
| rs11611651 | 12 | 133380790 | G | A | 0.0868 | -0.0271143 | 0.004525 | 2.05E-09 | 1232091 | 0.0001165 | 143.62 |
| rs13906 | 12 | 49952394 | C | T | 0.109 | 0.02452973 | 0.004088 | 1.98E-09 | 1232091 | 0.0001169 | 144.02 |
| rs4759229 | 12 | 56474480 | A | G | 0.656 | -0.0155696 | 0.002682 | 6.53E-09 | 1232091 | 0.0001094 | 134.81 |
| rs7134009 | 12 | 75263193 | T | C | 0.287 | 0.01579691 | 0.002884 | 4.3E-08 | 1174994 | 0.0001021 | 120.01 |
| rs7969559 | 12 | 69655167 | A | G | 0.713 | 0.01701588 | 0.002816 | 1.53E-09 | 1232091 | 0.0001185 | 146.02 |
| rs1108130 | 13 | 100648356 | T | A | 0.212 | -0.0239436 | 0.003117 | 1.57E-14 | 1232091 | 0.0001915 | 236.04 |
| rs12855717 | 13 | 101252635 | C | T | 0.538 | -0.0155237 | 0.002556 | 1.22E-09 | 1232091 | 0.0001198 | 147.62 |
| rs1413119 | 13 | 59339281 | C | T | 0.396318 | 0.01525509 | 0.002605 | 4.77E-09 | 1232091 | 0.0001114 | 137.22 |
| rs17197663 | 13 | 38172867 | G | A | 0.125 | 0.02158735 | 0.003852 | 2.06E-08 | 1232091 | 0.0001019 | 125.61 |
| rs1772572 | 13 | 81191176 | C | A | 0.324132 | 0.01686814 | 0.002722 | 5.62E-10 | 1232091 | 0.0001247 | 153.62 |
| rs3098272 | 13 | 55931424 | A | C | 0.798794 | 0.01780822 | 0.003178 | 2.08E-08 | 1232091 | 0.0001019 | 125.61 |
| rs4264267 | 13 | 38359676 | C | T | 0.527042 | -0.0147921 | 0.002552 | 6.82E-09 | 1232091 | 0.0001091 | 134.41 |
| rs4886207 | 13 | 60705792 | T | C | 0.637 | 0.01624668 | 0.00265 | 8.78E-10 | 1232091 | 0.0001221 | 150.42 |
| rs55786907 | 13 | 59871584 | A | G | 0.162492 | -0.0194452 | 0.003454 | 1.84E-08 | 1232091 | 0.0001029 | 126.81 |
| rs56367474 | 13 | 59454139 | C | T | 0.304 | 0.01729755 | 0.00277 | 4.2E-10 | 1232091 | 0.0001266 | 156.02 |
| rs61959481 | 13 | 55834929 | G | A | 0.21 | 0.0203442 | 0.003128 | 7.95E-11 | 1232091 | 0.0001373 | 169.22 |
| rs7333559 | 13 | 100546450 | G | A | 0.783 | 0.02321253 | 0.003091 | 5.94E-14 | 1232091 | 0.0001831 | 225.64 |
| rs75674569 | 13 | 96823724 | G | A | 0.0997 | 0.02533766 | 0.004253 | 2.58E-09 | 1232091 | 0.0001153 | 142.02 |
| rs9538162 | 13 | 59265043 | T | C | 0.415874 | -0.0173792 | 0.002585 | 1.76E-11 | 1232091 | 0.0001467 | 180.83 |
| rs9540731 | 13 | 66949370 | C | T | 0.509 | 0.01773032 | 0.002549 | 3.42E-12 | 1232091 | 0.0001571 | 193.63 |
| rs9545155 | 13 | 80191873 | T | C | 0.478 | 0.01607088 | 0.002551 | 3.04E-10 | 1232091 | 0.0001289 | 158.82 |
| rs12878369 | 14 | 28346502 | C | A | 0.414762 | -0.0174373 | 0.002588 | 1.6E-11 | 1230262 | 0.0001476 | 181.63 |
| rs1381287 | 14 | 98597552 | C | T | 0.467 | -0.0180166 | 0.002556 | 1.81E-12 | 1230262 | 0.0001616 | 198.83 |
| rs2145451 | 14 | 29316842 | T | C | 0.193 | 0.02004616 | 0.003231 | 5.44E-10 | 1230262 | 0.0001252 | 154.02 |
| rs2925128 | 14 | 98362355 | C | T | 0.385191 | -0.0168199 | 0.002683 | 3.67E-10 | 1173165 | 0.000134 | 157.22 |
| rs34940743 | 14 | 80102233 | A | G | 0.346 | -0.0159249 | 0.00268 | 2.8E-09 | 1230262 | 0.0001148 | 141.22 |
| rs55913542 | 14 | 99693843 | G | T | 0.175 | -0.0185623 | 0.003356 | 3.25E-08 | 1230262 | 9.949E-05 | 122.41 |
| rs8005334 | 14 | 79563654 | T | G | 0.36 | -0.0166734 | 0.002656 | 3.44E-10 | 1230262 | 0.0001281 | 157.62 |
| rs9323328 | 14 | 58653514 | A | G | 0.537 | 0.01423703 | 0.002557 | 2.55E-08 | 1230262 | 0.0001008 | 124.01 |
| rs12442563 | 15 | 83893243 | G | T | 0.223 | 0.02322961 | 0.003061 | 3.13E-14 | 1232091 | 0.000187 | 230.44 |
| rs1435672 | 15 | 36399479 | T | C | 0.56 | -0.0141051 | 0.002567 | 3.82E-08 | 1232091 | 9.804E-05 | 120.81 |
| rs1435741 | 15 | 47935843 | G | A | 0.432951 | -0.0183091 | 0.002571 | 1.09E-12 | 1232091 | 0.0001646 | 202.83 |
| rs2289791 | 15 | 67476952 | G | T | 0.247 | 0.01772552 | 0.002954 | 2.01E-09 | 1232091 | 0.0001169 | 144.02 |
| rs281296 | 15 | 47685010 | G | A | 0.357 | -0.0246892 | 0.002659 | 1.59E-20 | 1232091 | 0.0002798 | 344.90 |
| rs4310804 | 15 | 96858409 | C | G | 0.247 | 0.01818726 | 0.002954 | 7.55E-10 | 1232091 | 0.000123 | 151.62 |
| rs56902655 | 15 | 63898709 | T | G | 0.136 | 0.02186278 | 0.003717 | 4.09E-09 | 1232091 | 0.0001123 | 138.42 |
| rs60833441 | 15 | 74048768 | A | G | 0.461 | 0.01427664 | 0.002556 | 2.28E-08 | 1232091 | 0.0001013 | 124.81 |
| rs62007780 | 15 | 78025464 | G | T | 0.416 | 0.0159133 | 0.002585 | 7.48E-10 | 1232091 | 0.000123 | 151.62 |
| rs8027457 | 15 | 99204101 | T | C | 0.511 | -0.0153138 | 0.002549 | 1.88E-09 | 1232091 | 0.0001172 | 144.42 |
| rs1050847 | 16 | 87443734 | C | T | 0.559 | 0.01483002 | 0.002566 | 7.37E-09 | 1232091 | 0.0001084 | 133.61 |
| rs11076962 | 16 | 5811367 | T | C | 0.279 | -0.0182999 | 0.002841 | 1.2E-10 | 1232091 | 0.0001347 | 166.02 |
| rs1139897 | 16 | 720986 | G | A | 0.23 | 0.02408719 | 0.003028 | 1.77E-15 | 1232091 | 0.0002055 | 253.25 |
| rs11642231 | 16 | 89608702 | G | A | 0.369 | 0.01559834 | 0.00264 | 3.44E-09 | 1232091 | 0.0001133 | 139.62 |
| rs117657830 | 16 | 75766873 | A | G | 0.0417 | 0.03775969 | 0.006373 | 3.18E-09 | 1232091 | 0.000114 | 140.42 |
| rs12918191 | 16 | 50945156 | A | G | 0.243 | 0.01972704 | 0.002971 | 3.14E-11 | 1232091 | 0.0001432 | 176.42 |
| rs4785187 | 16 | 49766772 | G | A | 0.223 | -0.0199772 | 0.003061 | 6.55E-11 | 1232091 | 0.0001383 | 170.42 |
| rs4788676 | 16 | 72950468 | T | C | 0.228528 | 0.01774504 | 0.003034 | 4.92E-09 | 1232091 | 0.000111 | 136.81 |
| rs62052916 | 16 | 72574550 | A | T | 0.0701 | 0.03191377 | 0.00499 | 1.62E-10 | 1232091 | 0.0001328 | 163.62 |
| rs6497840 | 16 | 25351633 | G | A | 0.707 | -0.0227703 | 0.002867 | 2.01E-15 | 1174994 | 0.0002148 | 252.45 |
| rs7188873 | 16 | 24727064 | A | G | 0.612998 | -0.0202958 | 0.002616 | 8.46E-15 | 1232091 | 0.0001954 | 240.85 |
| rs7192140 | 16 | 10173748 | T | C | 0.498 | 0.01688338 | 0.002548 | 3.4E-11 | 1232091 | 0.0001425 | 175.62 |
| rs8050598 | 16 | 49891964 | C | T | 0.254117 | -0.0186698 | 0.002926 | 1.76E-10 | 1232091 | 0.0001321 | 162.82 |
| rs9302604 | 16 | 69576894 | A | G | 0.435 | -0.0187095 | 0.00257 | 3.29E-13 | 1232091 | 0.0001721 | 212.04 |
| rs9922607 | 16 | 17570220 | C | T | 0.2 | 0.02215931 | 0.003185 | 3.42E-12 | 1232091 | 0.0001571 | 193.63 |
| rs9936784 | 16 | 72230694 | T | G | 0.53418 | -0.0139895 | 0.002554 | 4.33E-08 | 1232091 | 9.74E-05 | 120.01 |
| rs9941217 | 16 | 18050926 | C | G | 0.352204 | 0.01855667 | 0.002667 | 3.5E-12 | 1232091 | 0.0001571 | 193.63 |
| rs11078713 | 17 | 7795972 | A | G | 0.419348 | 0.01458289 | 0.002582 | 1.59E-08 | 1232091 | 0.0001036 | 127.61 |
| rs11651955 | 17 | 16235462 | G | A | 0.499 | 0.01402639 | 0.002548 | 3.74E-08 | 1232091 | 9.837E-05 | 121.21 |
| rs17692129 | 17 | 44793283 | C | T | 0.331 | -0.0195989 | 0.002707 | 4.57E-13 | 1232091 | 0.0001701 | 209.64 |
| rs2344976 | 17 | 30685935 | T | C | 0.612 | 0.01508774 | 0.002615 | 7.98E-09 | 1232091 | 0.0001081 | 133.21 |
| rs2587507 | 17 | 77790135 | T | C | 0.502 | 0.01466024 | 0.002548 | 8.69E-09 | 1232091 | 0.0001075 | 132.41 |
| rs28441558 | 17 | 7803118 | T | C | 0.0563 | 0.03556498 | 0.005527 | 1.24E-10 | 1232091 | 0.0001344 | 165.62 |
| rs2938134 | 17 | 50243397 | C | A | 0.673 | 0.01750102 | 0.002781 | 3.14E-10 | 1174994 | 0.0001348 | 158.42 |
| rs3764351 | 17 | 37824339 | G | A | 0.657 | 0.01474916 | 0.002684 | 3.89E-08 | 1232091 | 9.804E-05 | 120.81 |
| rs4790874 | 17 | 1995177 | C | T | 0.532 | -0.0174491 | 0.002553 | 8.43E-12 | 1232091 | 0.0001516 | 186.83 |
| rs67777803 | 17 | 27323322 | G | T | 0.172 | 0.02460149 | 0.003376 | 3.18E-13 | 1232091 | 0.0001724 | 212.44 |
| rs72836318 | 17 | 44121579 | T | C | 0.246 | 0.01712233 | 0.002958 | 7E-09 | 1232091 | 0.0001088 | 134.01 |
| rs75919030 | 17 | 50193197 | T | C | 0.267 | 0.0209664 | 0.00288 | 3.35E-13 | 1232091 | 0.0001721 | 212.04 |
| rs11872397 | 18 | 72535282 | G | A | 0.253 | 0.01711395 | 0.002931 | 5.2E-09 | 1232091 | 0.0001107 | 136.41 |
| rs1373178 | 18 | 49967811 | T | G | 0.588 | 0.0203164 | 0.002589 | 4.16E-15 | 1232091 | 0.0002 | 246.45 |
| rs34342129 | 18 | 5872472 | T | C | 0.509 | 0.01428101 | 0.002549 | 2.13E-08 | 1232091 | 0.0001019 | 125.61 |
| rs4476253 | 18 | 25253297 | G | A | 0.24 | 0.01848616 | 0.002983 | 5.78E-10 | 1232091 | 0.0001247 | 153.62 |
| rs62098013 | 18 | 50863861 | G | A | 0.365322 | -0.01771 | 0.002646 | 2.24E-11 | 1232091 | 0.0001454 | 179.23 |
| rs67050670 | 18 | 39297254 | A | G | 0.229 | 0.02027227 | 0.003032 | 2.34E-11 | 1232091 | 0.0001451 | 178.83 |
| rs71367544 | 18 | 77574374 | C | T | 0.203 | -0.0205522 | 0.003167 | 8.54E-11 | 1232091 | 0.0001367 | 168.42 |
| rs72898831 | 18 | 42658643 | A | G | 0.155 | 0.02441586 | 0.00352 | 4.14E-12 | 1232091 | 0.0001562 | 192.43 |
| rs7505855 | 18 | 31696075 | C | T | 0.586 | 0.01698178 | 0.002587 | 5.31E-11 | 1232091 | 0.0001399 | 172.42 |
| rs8083764 | 18 | 49874515 | G | T | 0.306171 | 0.01595172 | 0.002764 | 7.97E-09 | 1232091 | 0.0001081 | 133.21 |
| rs8096225 | 18 | 36921851 | A | C | 0.703 | -0.0155245 | 0.002788 | 2.63E-08 | 1232091 | 0.0001006 | 124.01 |
| rs10853981 | 19 | 4965064 | G | A | 0.33036 | -0.0147872 | 0.002709 | 4.88E-08 | 1232091 | 9.675E-05 | 119.21 |
| rs1126757 | 19 | 55879872 | C | T | 0.473 | -0.0141623 | 0.002552 | 2.92E-08 | 1232091 | 9.999E-05 | 123.21 |
| rs113230003 | 19 | 18460956 | G | A | 0.255 | 0.01887615 | 0.002923 | 1.05E-10 | 1232091 | 0.0001354 | 166.82 |
| rs76608582 | 19 | 4474725 | C | A | 0.0489 | 0.03454929 | 0.005908 | 4.88E-09 | 1232091 | 0.000111 | 136.81 |
| rs8103660 | 19 | 18566395 | T | C | 0.354431 | -0.0158026 | 0.002664 | 3.03E-09 | 1232091 | 0.0001143 | 140.82 |
| rs3810496 | 20 | 62406886 | T | C | 0.619436 | -0.0158813 | 0.002629 | 1.54E-09 | 1227798 | 0.0001189 | 146.02 |
| rs6011779 | 20 | 61984317 | C | T | 0.806 | 0.01917655 | 0.003228 | 2.83E-09 | 1227798 | 0.000115 | 141.22 |
| rs6050446 | 20 | 25195509 | A | G | 0.971 | -0.0544098 | 0.007611 | 8.8E-13 | 1225969 | 0.0001667 | 204.43 |
| rs6058782 | 20 | 29946968 | C | T | 0.908 | -0.0297103 | 0.004419 | 1.78E-11 | 1225969 | 0.0001475 | 180.83 |
| rs6073075 | 20 | 42015801 | T | A | 0.824 | 0.01870404 | 0.003354 | 2.44E-08 | 1225969 | 0.0001015 | 124.41 |
| rs910912 | 20 | 54462393 | T | C | 0.739 | 0.01676993 | 0.002906 | 7.82E-09 | 1227798 | 0.0001085 | 133.21 |
| rs4818005 | 21 | 40588819 | G | A | 0.581 | 0.02043098 | 0.002644 | 1.09E-14 | 1174994 | 0.0002032 | 238.85 |
| rs139896 | 22 | 38397797 | T | C | 0.648 | -0.0154404 | 0.002668 | 7.14E-09 | 1232091 | 0.0001088 | 134.01 |
| rs4822102 | 22 | 42698430 | C | T | 0.618 | 0.01654281 | 0.002622 | 2.78E-10 | 1232091 | 0.0001292 | 159.22 |
| rs9627272 | 22 | 46442288 | G | C | 0.407 | 0.0154737 | 0.002593 | 2.42E-09 | 1232091 | 0.0001156 | 142.42 |

SNP: single nucleotide polymorphism; EA: effect allele; OA: other allele; SE: standard error; R2: proportion of phenotypic variance explained by SNPs.

**Supplementary Table 2. Main characteristics of SNPs to instrument alcoholic drinks per week.**

| **SNP** | **Chr** | **Position** | **EA** | **OA** | **EAF** | **Beta** | **SE** | **P value** | **Sample size** | **R^2^** | **F** |
| --- | --- | --- | --- | --- | --- | --- | --- | --- | --- | --- | --- |
| rs10753661 | 1 | 1.65E+08 | A | G | 0.684 | 0.008638 | 0.001569 | 3.76E-08 | 939356 | 3.22561E-05 | 30.30 |
| rs12088813 | 1 | 66407700 | C | A | 0.267 | 0.009329 | 0.001649 | 1.58E-08 | 939356 | 3.40659E-05 | 32.00 |
| rs28680958 | 1 | 1.74E+08 | A | G | 0.217 | 0.010996 | 0.00177 | 5.13E-10 | 939356 | 4.1092E-05 | 38.60 |
| rs5024204 | 1 | 71491890 | T | A | 0.278 | -0.0097 | 0.001628 | 2.55E-09 | 939356 | 3.77918E-05 | 35.50 |
| rs58107686 | 1 | 33837334 | A | C | 0.328 | 0.009747 | 0.001585 | 7.79E-10 | 902538 | 4.18819E-05 | 37.80 |
| rs705687 | 1 | 4548453 | G | A | 0.785 | 0.010904 | 0.001776 | 8.15E-10 | 939356 | 4.01339E-05 | 37.70 |
| rs823114 | 1 | 2.06E+08 | A | G | 0.553 | -0.00877 | 0.001467 | 2.31E-09 | 939356 | 3.80048E-05 | 35.70 |
| rs1004787 | 2 | 45159091 | A | G | 0.550918 | -0.00844 | 0.001465 | 8.40E-09 | 941280 | 3.52711E-05 | 33.20 |
| rs11692435 | 2 | 98275354 | A | G | 0.0852 | -0.01745 | 0.002616 | 2.53E-11 | 937516 | 4.74659E-05 | 44.50 |
| rs1260326 | 2 | 27730940 | C | T | 0.601 | -0.02089 | 0.001488 | 8.05E-45 | 941280 | 0.000209289 | 197.04 |
| rs13024996 | 2 | 1.44E+08 | A | C | 0.364 | 0.010913 | 0.001515 | 5.72E-13 | 941280 | 5.51377E-05 | 51.90 |
| rs13032049 | 2 | 63581507 | G | A | 0.283 | -0.01019 | 0.001618 | 3.00E-10 | 941280 | 4.21766E-05 | 39.70 |
| rs13383034 | 2 | 45155276 | T | C | 0.329 | -0.01493 | 0.001551 | 6.31E-22 | 941280 | 9.83767E-05 | 92.61 |
| rs2178197 | 2 | 27860551 | G | A | 0.569083 | 0.008781 | 0.001472 | 2.45E-09 | 941280 | 3.78208E-05 | 35.60 |
| rs56337305 | 2 | 2.25E+08 | C | T | 0.383 | 0.009588 | 0.001499 | 1.63E-10 | 941280 | 4.34515E-05 | 40.90 |
| rs72859280 | 2 | 1.48E+08 | T | G | 0.0362 | -0.02289 | 0.003902 | 4.44E-09 | 941280 | 3.6546E-05 | 34.40 |
| rs77165542 | 2 | 430975 | T | C | 0.0349 | 0.026011 | 0.003971 | 5.63E-11 | 941280 | 4.55762E-05 | 42.90 |
| rs828867 | 2 | 74334462 | A | G | 0.545 | -0.00876 | 0.001464 | 2.15E-09 | 941280 | 3.80333E-05 | 35.80 |
| rs13066454 | 3 | 93994255 | T | C | 0.398 | 0.008776 | 0.001492 | 4.13E-09 | 937516 | 3.6906E-05 | 34.60 |
| rs13094887 | 3 | 70968431 | T | A | 0.301 | 0.01031 | 0.001589 | 8.57E-11 | 941280 | 4.47263E-05 | 42.10 |
| rs2011092 | 3 | 1.41E+08 | C | T | 0.338643 | 0.0089 | 0.00154 | 7.35E-09 | 941280 | 3.54836E-05 | 33.40 |
| rs60654199 | 3 | 1.41E+08 | A | C | 0.0629 | 0.01666 | 0.003002 | 2.85E-08 | 941280 | 3.27214E-05 | 30.80 |
| rs62250685 | 3 | 85457240 | G | A | 0.614 | 0.014357 | 0.0015 | 1.05E-21 | 937516 | 9.7705E-05 | 91.61 |
| rs6787172 | 3 | 1.58E+08 | G | T | 0.554 | 0.008031 | 0.001466 | 4.27E-08 | 941280 | 3.18715E-05 | 30.00 |
| rs9838144 | 3 | 1.32E+08 | C | G | 0.209 | 0.009964 | 0.001793 | 2.65E-08 | 941280 | 3.28276E-05 | 30.90 |
| rs10004020 | 4 | 1.53E+08 | A | G | 0.72 | -0.00905 | 0.001623 | 2.43E-08 | 941280 | 3.30401E-05 | 31.10 |
| rs10028756 | 4 | 1E+08 | A | G | 0.129457 | 0.018575 | 0.002171 | 1.16E-17 | 941280 | 7.77664E-05 | 73.21 |
| rs1154414 | 4 | 1E+08 | C | T | 0.141063 | -0.01763 | 0.002094 | 3.74E-17 | 941280 | 7.5323E-05 | 70.91 |
| rs11940694 | 4 | 39414993 | G | A | 0.597 | -0.02595 | 0.001486 | 3.03E-68 | 941280 | 0.000324027 | 305.10 |
| rs1229984 | 4 | 1E+08 | C | T | 0.963 | -0.15053 | 0.003861 | <2.2e-308 | 941280 | 0.001614822 | 1522.46 |
| rs12499107 | 4 | 99678691 | G | A | 0.131202 | -0.01266 | 0.002159 | 4.45E-09 | 941280 | 3.6546E-05 | 34.40 |
| rs13107325 | 4 | 1.03E+08 | T | C | 0.0722 | 0.027505 | 0.002816 | 1.53E-22 | 941280 | 0.000101351 | 95.41 |
| rs144198753 | 4 | 99713350 | T | C | 0.016315 | 0.041827 | 0.005903 | 1.35E-12 | 893955 | 5.6155E-05 | 50.20 |
| rs17029090 | 4 | 1E+08 | G | A | 0.019896 | 0.049127 | 0.005219 | 4.75E-21 | 941280 | 9.41271E-05 | 88.61 |
| rs2165670 | 4 | 1E+08 | A | G | 0.106337 | -0.02308 | 0.002364 | 1.67E-22 | 941280 | 0.000101245 | 95.31 |
| rs35538052 | 4 | 39418965 | A | G | 0.379 | 0.008525 | 0.001502 | 1.39E-08 | 941280 | 3.42087E-05 | 32.20 |
| rs36052336 | 4 | 1E+08 | G | A | 0.061496 | 0.018429 | 0.003034 | 1.23E-09 | 941280 | 3.92019E-05 | 36.90 |
| rs3748034 | 4 | 3446091 | T | G | 0.143 | 0.01174 | 0.002082 | 1.67E-08 | 941280 | 3.37838E-05 | 31.80 |
| rs4690727 | 4 | 1.44E+08 | G | C | 0.718 | -0.01082 | 0.00162 | 2.43E-11 | 941280 | 4.73823E-05 | 44.60 |
| rs4699791 | 4 | 1.01E+08 | A | G | 0.095726 | -0.01857 | 0.002477 | 6.58E-14 | 941280 | 5.97059E-05 | 56.20 |
| rs79139602 | 4 | 1E+08 | T | A | 0.021061 | -0.06027 | 0.005076 | 1.80E-32 | 941280 | 0.000149796 | 141.02 |
| rs11739827 | 5 | 1.67E+08 | T | G | 0.451 | 0.008375 | 0.001469 | 1.18E-08 | 935645 | 3.47354E-05 | 32.50 |
| rs12655091 | 5 | 1.44E+08 | A | G | 0.53 | 0.008312 | 0.00146 | 1.25E-08 | 941280 | 3.44212E-05 | 32.40 |
| rs4916723 | 5 | 87854395 | C | A | 0.416 | 0.009952 | 0.001479 | 1.72E-11 | 941280 | 4.8126E-05 | 45.30 |
| rs55872084 | 5 | 1.56E+08 | T | G | 0.235 | -0.00998 | 0.001719 | 6.32E-09 | 941280 | 3.58023E-05 | 33.70 |
| rs10085696 | 7 | 69783020 | G | A | 0.186 | 0.011409 | 0.001873 | 1.12E-09 | 941280 | 3.94144E-05 | 37.10 |
| rs10236149 | 7 | 98977515 | G | A | 0.123 | 0.013498 | 0.002219 | 1.18E-09 | 941280 | 3.93082E-05 | 37.00 |
| rs35034355 | 7 | 1.04E+08 | A | G | 0.521 | 0.008097 | 0.001459 | 2.87E-08 | 941280 | 3.27214E-05 | 30.80 |
| rs6460047 | 7 | 73042443 | C | T | 0.208 | -0.01162 | 0.001796 | 9.69E-11 | 941280 | 4.45139E-05 | 41.90 |
| rs1217091 | 8 | 64527399 | C | T | 0.812 | -0.01216 | 0.001865 | 7.05E-11 | 941280 | 4.51513E-05 | 42.50 |
| rs13250583 | 8 | 20949917 | T | C | 0.213 | 0.009718 | 0.00178 | 4.70E-08 | 941280 | 3.1659E-05 | 29.80 |
| rs28601761 | 8 | 1.27E+08 | G | C | 0.42 | -0.0091 | 0.001477 | 7.17E-10 | 941280 | 4.03706E-05 | 38.00 |
| rs10978550 | 9 | 1.09E+08 | C | T | 0.206 | 0.011748 | 0.001802 | 7.15E-11 | 941280 | 4.51513E-05 | 42.50 |
| rs55932213 | 9 | 1.09E+08 | G | A | 0.736387 | -0.00949 | 0.001654 | 9.55E-09 | 941280 | 3.49524E-05 | 32.90 |
| rs17665139 | 10 | 1.25E+08 | T | C | 0.149 | 0.01156 | 0.002047 | 1.59E-08 | 941280 | 3.389E-05 | 31.90 |
| rs7074871 | 10 | 1.11E+08 | A | G | 0.255 | 0.0094 | 0.001672 | 1.86E-08 | 941280 | 3.35713E-05 | 31.60 |
| rs10750025 | 11 | 1.13E+08 | T | C | 0.686 | -0.01032 | 0.00157 | 4.89E-11 | 941280 | 4.5895E-05 | 43.20 |
| rs11030084 | 11 | 27643725 | T | C | 0.184 | 0.010607 | 0.001881 | 1.72E-08 | 941280 | 3.37838E-05 | 31.80 |
| rs12795042 | 11 | 1.34E+08 | C | A | 0.623 | 0.008319 | 0.001504 | 3.25E-08 | 941280 | 3.25089E-05 | 30.60 |
| rs1713676 | 11 | 1.14E+08 | G | A | 0.522454 | 0.007992 | 0.001459 | 4.29E-08 | 941280 | 3.18715E-05 | 30.00 |
| rs4938230 | 11 | 1.16E+08 | A | C | 0.842 | -0.01281 | 0.001998 | 1.48E-10 | 941280 | 4.36639E-05 | 41.10 |
| rs56030824 | 11 | 47397353 | A | G | 0.322 | 0.011602 | 0.001563 | 1.15E-13 | 937516 | 5.87723E-05 | 55.10 |
| rs682011 | 11 | 1.22E+08 | C | T | 0.559 | -0.00821 | 0.001468 | 2.22E-08 | 941280 | 3.32526E-05 | 31.30 |
| rs7950166 | 11 | 8642218 | T | C | 0.637 | 0.009799 | 0.001516 | 9.89E-11 | 941280 | 4.44076E-05 | 41.80 |
| rs10506274 | 12 | 81601464 | T | G | 0.484 | 0.009037 | 0.001458 | 5.78E-10 | 941280 | 4.07955E-05 | 38.40 |
| rs10876188 | 12 | 51895882 | T | C | 0.457 | 0.007987 | 0.001463 | 4.84E-08 | 941280 | 3.1659E-05 | 29.80 |
| rs3809162 | 12 | 54674235 | G | A | 0.397 | -0.00906 | 0.00149 | 1.19E-09 | 941280 | 3.93082E-05 | 37.00 |
| rs500321 | 13 | 27124360 | T | A | 0.736 | 0.009669 | 0.001653 | 4.92E-09 | 941280 | 3.63335E-05 | 34.20 |
| rs1123285 | 14 | 57274519 | G | C | 0.335 | 0.008897 | 0.001544 | 8.14E-09 | 941280 | 3.52711E-05 | 33.20 |
| rs11625650 | 14 | 1.05E+08 | A | G | 0.233 | 0.009568 | 0.001724 | 2.89E-08 | 941280 | 3.27214E-05 | 30.80 |
| rs2180870 | 14 | 58782779 | C | T | 0.135 | 0.012178 | 0.002133 | 1.12E-08 | 941280 | 3.46337E-05 | 32.60 |
| rs28929474 | 14 | 94844947 | T | C | 0.0183 | 0.0368 | 0.005438 | 1.34E-11 | 941280 | 4.86571E-05 | 45.80 |
| rs12907323 | 15 | 86796012 | G | A | 0.411 | -0.0085 | 0.001481 | 9.93E-09 | 941280 | 3.49524E-05 | 32.90 |
| rs2472297 | 15 | 75027880 | T | C | 0.249 | -0.01061 | 0.001685 | 3.10E-10 | 941280 | 4.20704E-05 | 39.60 |
| rs113443718 | 16 | 29892184 | A | G | 0.305 | 0.010208 | 0.001585 | 1.19E-10 | 939356 | 4.41792E-05 | 41.50 |
| rs17177078 | 16 | 24810681 | T | C | 0.0626 | 0.022315 | 0.003012 | 1.27E-13 | 939356 | 5.84443E-05 | 54.90 |
| rs2764771 | 16 | 20013793 | A | G | 0.307 | -0.00989 | 0.001582 | 4.02E-10 | 939356 | 4.16243E-05 | 39.10 |
| rs62044525 | 16 | 64872590 | G | C | 0.184 | 0.012173 | 0.001883 | 1.03E-10 | 939356 | 4.44986E-05 | 41.80 |
| rs7185555 | 16 | 69131281 | C | G | 0.153 | 0.011101 | 0.002027 | 4.24E-08 | 939356 | 3.19368E-05 | 30.00 |
| rs79616692 | 16 | 72338507 | C | G | 0.108 | -0.0163 | 0.002351 | 4.11E-12 | 939356 | 5.12053E-05 | 48.10 |
| rs10438820 | 17 | 78524597 | T | C | 0.702 | -0.00897 | 0.001593 | 1.76E-08 | 941280 | 3.36775E-05 | 31.70 |
| rs2854334 | 17 | 29715500 | G | A | 0.615 | -0.00922 | 0.001498 | 7.51E-10 | 941280 | 4.02643E-05 | 37.90 |
| rs3803800 | 17 | 7462969 | G | A | 0.786 | -0.01138 | 0.001777 | 1.50E-10 | 941280 | 4.35577E-05 | 41.00 |
| rs4548913 | 17 | 2209888 | A | G | 0.632 | 0.00836 | 0.001511 | 3.11E-08 | 941280 | 3.25089E-05 | 30.60 |
| rs4092465 | 18 | 55080437 | G | A | 0.635 | 0.008292 | 0.001514 | 4.39E-08 | 941280 | 3.18715E-05 | 30.00 |
| rs9950000 | 18 | 53052169 | T | C | 0.395 | 0.009118 | 0.001491 | 9.38E-10 | 941280 | 3.97331E-05 | 37.40 |
| rs281379 | 19 | 49214274 | A | G | 0.508 | -0.01372 | 0.001458 | 4.91E-21 | 941280 | 9.41271E-05 | 88.61 |
| rs4815364 | 20 | 25035711 | A | G | 0.616 | -0.00858 | 0.001499 | 1.02E-08 | 941280 | 3.48462E-05 | 32.80 |
| rs9607814 | 22 | 41946519 | A | C | 0.2 | 0.010181 | 0.001859 | 4.31E-08 | 904462 | 3.31689E-05 | 30.00 |

SNP: single nucleotide polymorphism; EA: effect allele; OA: other allele; SE: standard error; R2: proportion of phenotypic variance explained by SNPs.

**Supplementary Table 3. Main characteristics of SNPs to instrument coffee consumption.**

| **SNP** | **Chr** | **Position** | **EA** | **OA** | **EAF** | **Beta** | **SE** | **P value** | **Sample size** | **R^2^** | **F** |
| --- | --- | --- | --- | --- | --- | --- | --- | --- | --- | --- | --- |
| rs574367 | 1 | 177873210 | T | G | 0.19 | 1.05 | 0.18 | 8.06E-09 | 375833 | 9.05E-05 | 34.03 |
| rs10865548 | 2 | 631606 | G | A | 0.21 | 1.54 | 0.19 | 4.46E-15 | 375833 | 0.000175 | 65.69 |
| rs1260326 | 2 | 27730940 | C | T | 0.16 | 1.36 | 0.15 | 2.62E-19 | 375833 | 0.000219 | 82.20 |
| rs1057868 | 7 | 75615006 | T | C | 0.17 | 1.97 | 0.16 | 5.26E-33 | 375833 | 0.000403 | 151.60 |
| rs117692895 | 7 | 17277692 | C | G | 0.71 | 4.29 | 0.67 | 4.13E-10 | 374046 | 0.00011 | 41.00 |
| rs34060476 | 7 | 73037956 | G | A | 0.23 | 1.89 | 0.22 | 5.06E-18 | 375833 | 0.000196 | 73.80 |
| rs4410790 | 7 | 17284577 | C | T | 0.16 | 3.94 | 0.15 | 5.6E-141 | 375833 | 0.001832 | 689.93 |
| rs4719497 | 7 | 17334899 | T | C | 0.23 | 1.2 | 0.22 | 4.23E-08 | 375833 | 7.92E-05 | 29.75 |
| rs73073176 | 7 | 17562952 | C | T | 0.23 | 2.31 | 0.22 | 5.56E-25 | 375833 | 0.000293 | 110.25 |
| rs597045 | 11 | 56272114 | A | T | 0.17 | 1.07 | 0.16 | 6.62E-11 | 375833 | 0.000119 | 44.72 |
| rs1956218 | 14 | 33075243 | G | A | 0.16 | 0.82 | 0.15 | 3.62E-08 | 375833 | 7.95E-05 | 29.88 |
| rs2472297 | 15 | 75027880 | T | C | 0.18 | 4.54 | 0.17 | 5.2E-155 | 375833 | 0.001894 | 713.20 |
| rs66723169 | 18 | 57808978 | A | C | 0.19 | 1.47 | 0.18 | 9.88E-17 | 375833 | 0.000177 | 66.69 |
| rs2330783 | 22 | 24747031 | G | T | 0.67 | 4.53 | 0.63 | 1.57E-12 | 375833 | 0.000138 | 51.70 |

SNP: single nucleotide polymorphism; EA: effect allele; OA: other allele; SE: standard error; R2: proportion of phenotypic variance explained by SNPs.

**Supplementary Table 4. Main characteristics of SNPs to instrument moderate-to-vigorous physical activity.**

| **SNP** | **Chr** | **Position** | **EA** | **OA** | **EAF** | **Beta** | **SE** | **P value** | **Sample size** | **R^2^** | **F** |
| --- | --- | --- | --- | --- | --- | --- | --- | --- | --- | --- | --- |
| rs2942127 | 1 | 204420067 | A | G | 0.824644 | -0.01604 | 0.002903 | 3.29997E-08 | 377234 | 7.44E-05 | 28.06 |
| rs1974771 | 2 | 54278543 | A | G | 0.099975 | 0.021339 | 0.003678 | 6.59994E-09 | 377234 | 8.19E-05 | 30.91 |
| rs2035562 | 3 | 85056521 | G | A | 0.672483 | 0.013876 | 0.002356 | 3.89996E-09 | 377234 | 8.48E-05 | 32.00 |
| rs2114286 | 3 | 41194283 | G | A | 0.534243 | 0.012245 | 0.002217 | 3.29997E-08 | 377234 | 7.46E-05 | 28.15 |
| rs877483 | 3 | 53846741 | C | T | 0.566815 | -0.01223 | 0.002228 | 0.00000004 | 377234 | 7.34E-05 | 27.70 |
| rs1972763 | 4 | 159860563 | T | C | 0.657628 | -0.01284 | 0.002324 | 3.29997E-08 | 377234 | 7.42E-05 | 28.00 |
| rs77742115 | 5 | 18330424 | C | T | 0.138319 | 0.018348 | 0.003198 | 9.59997E-09 | 377234 | 8.02E-05 | 30.27 |
| rs1043595 | 7 | 128410012 | A | G | 0.282865 | -0.01441 | 0.002454 | 4.30002E-09 | 377234 | 8.43E-05 | 31.79 |
| rs1186721 | 7 | 34974602 | A | G | 0.315844 | 0.01299 | 0.002372 | 4.39997E-08 | 377234 | 7.29E-05 | 27.51 |
| rs7804463 | 7 | 133447651 | C | T | 0.470424 | -0.01501 | 0.002213 | 1.20005E-11 | 377234 | 0.000112 | 42.35 |
| rs921915 | 7 | 50228581 | C | T | 0.587905 | 0.013888 | 0.00224 | 5.69994E-10 | 377234 | 9.35E-05 | 35.26 |
| rs2988004 | 9 | 37044388 | G | T | 0.442245 | 0.013171 | 0.00224 | 4.09996E-09 | 377234 | 8.56E-05 | 32.29 |
| rs7326482 | 13 | 54037803 | T | G | 0.615163 | 0.012961 | 0.002294 | 0.000000016 | 377234 | 7.95E-05 | 30.00 |
| rs10145335 | 14 | 98547748 | A | G | 0.250611 | 0.014122 | 0.002541 | 2.69998E-08 | 377234 | 7.49E-05 | 28.26 |
| rs12912808 | 15 | 95292223 | T | C | 0.148607 | -0.01755 | 0.003109 | 0.000000017 | 377234 | 7.79E-05 | 29.39 |
| rs4886868 | 15 | 74353561 | G | T | 0.585862 | 0.012495 | 0.002266 | 3.50002E-08 | 377234 | 7.58E-05 | 28.58 |
| rs429358 | 19 | 45411941 | C | T | 0.154172 | 0.021982 | 0.003054 | 6.09958E-13 | 377234 | 0.000126 | 47.55 |
| rs1921981 | 21 | 42422547 | A | G | 0.325647 | -0.01304 | 0.002371 | 3.79997E-08 | 377234 | 7.46E-05 | 28.16 |

SNP: single nucleotide polymorphism; EA: effect allele; OA: other allele; SE: standard error; R2: proportion of phenotypic variance explained by SNPs.

**Supplementary Table 5. Main characteristics of SNPs to instrument sedentary behavior.**

| **SNP** | **Chr** | **Position** | **EA** | **OA** | **EAF** | **Beta** | **SE** | **P value** | **Sample size** | **R^2^** | **F** |
| --- | --- | --- | --- | --- | --- | --- | --- | --- | --- | --- | --- |
| rs61776614 | 1 | 2166406 | C | T | 0.925 | 0.05 | 0.009 | 4.7E-08 | 91105 | 0.000347 | 31.61 |
| rs1858242 | 3 | 68527135 | A | G | 0.259 | 0.031 | 0.005 | 3.1E-09 | 91105 | 0.000369 | 33.62 |
| rs26579 | 5 | 87985295 | G | C | 0.415 | 0.028 | 0.005 | 2.6E-09 | 91105 | 0.000381 | 34.69 |
| rs6870096 | 5 | 151945811 | C | G | 0.321 | -0.028 | 0.005 | 2.3E-08 | 91105 | 0.000342 | 31.15 |

SNP: single nucleotide polymorphism; EA: effect allele; OA: other allele; SE: standard error; R2: proportion of phenotypic variance explained by SNPs.

**Supplementary Table 6. Main characteristics of SNPs to instrument insomnia.**

| **SNP** | **Chr** | **Position** | **EA** | **OA** | **EAF** | **Beta** | **SE** | **P value** | **Sample size** | **R^2^** | **F** |
| --- | --- | --- | --- | --- | --- | --- | --- | --- | --- | --- | --- |
| rs10800992 | 1 | 190900576 | T | C | 0.4431 | 0.018284 | 0.006 | 3.835E-12 | 1331010 | 0.000165 | 219.64 |
| rs11119409 | 1 | 210293333 | T | C | 0.5866 | -0.01502 | 0.006 | 1.193E-08 | 1331010 | 0.000109 | 145.71 |
| rs11588755 | 1 | 57819204 | A | G | 0.522 | -0.01502 | 0.006 | 5.142E-09 | 1331010 | 0.000113 | 149.92 |
| rs11803128 | 1 | 190060095 | A | G | 0.6541 | -0.01773 | 0.006 | 6.852E-11 | 1331010 | 0.000142 | 189.33 |
| rs12030482 | 1 | 96961268 | A | T | 0.2201 | 0.017868 | 0.007 | 8.161E-09 | 1331010 | 0.00011 | 145.90 |
| rs1289939 | 1 | 117944435 | T | C | 0.2325 | -0.01773 | 0.007 | 5.995E-09 | 1331010 | 0.000112 | 149.32 |
| rs1620977 | 1 | 72729142 | A | G | 0.2696 | 0.022428 | 0.007 | 2.272E-14 | 1331010 | 0.000198 | 263.74 |
| rs1937447 | 1 | 66358242 | C | G | 0.7593 | -0.01682 | 0.007 | 2.075E-08 | 1331010 | 0.000103 | 137.74 |
| rs2089358 | 1 | 37194103 | T | C | 0.7038 | -0.01773 | 0.007 | 2.745E-10 | 1331010 | 0.000131 | 174.45 |
| rs2815757 | 1 | 72764289 | T | C | 0.8089 | 0.024075 | 0.008 | 2.238E-13 | 1331010 | 0.000179 | 238.55 |
| rs5877 | 1 | 173878862 | T | C | 0.6691 | 0.015779 | 0.006 | 1.228E-08 | 1331010 | 0.00011 | 146.75 |
| rs61765555 | 1 | 73957815 | T | C | 0.2552 | -0.01954 | 0.007 | 3.999E-11 | 1331010 | 0.000145 | 193.26 |
| rs623025 | 1 | 201765094 | T | C | 0.2552 | -0.01637 | 0.007 | 3.159E-08 | 1331010 | 0.000102 | 135.67 |
| rs6702604 | 1 | 107190062 | A | G | 0.5843 | -0.01592 | 0.006 | 1.303E-09 | 1331010 | 0.000123 | 163.96 |
| rs699844 | 1 | 74878253 | A | G | 0.9195 | 0.026125 | 0.011 | 4.11E-08 | 1331010 | 0.000101 | 134.49 |
| rs1064213 | 2 | 198950240 | A | G | 0.4789 | -0.01592 | 0.006 | 6.407E-10 | 1331010 | 0.000127 | 168.45 |
| rs10928256 | 2 | 146458738 | T | C | 0.4192 | 0.01494 | 0.006 | 1.614E-08 | 1331010 | 0.000109 | 144.69 |
| rs113851554 | 2 | 66750564 | T | G | 0.0506 | 0.089552 | 0.014 | 1.563E-51 | 1331010 | 0.000771 | 1026.35 |
| rs116466468 | 2 | 159137557 | T | C | 0.7593 | 0.019116 | 0.007 | 2.106E-10 | 1331010 | 0.000134 | 177.81 |
| rs11679943 | 2 | 77724624 | A | G | 0.3471 | 0.016197 | 0.006 | 3.155E-09 | 1331010 | 0.000119 | 158.29 |
| rs12614369 | 2 | 66792109 | A | G | 0.8158 | 0.019116 | 0.008 | 7.214E-09 | 1331010 | 0.00011 | 146.20 |
| rs13010288 | 2 | 51824512 | T | G | 0.1326 | -0.02595 | 0.009 | 9.257E-12 | 1331010 | 0.000155 | 206.20 |
| rs1519102 | 2 | 66677816 | C | G | 0.6891 | -0.01592 | 0.006 | 1.903E-08 | 1331010 | 0.000109 | 144.61 |
| rs1530938 | 2 | 236900633 | A | G | 0.4423 | 0.015779 | 0.006 | 8.823E-10 | 1331010 | 0.000123 | 163.50 |
| rs1861412 | 2 | 58893065 | A | G | 0.4338 | 0.016616 | 0.006 | 1.668E-10 | 1331010 | 0.000136 | 180.53 |
| rs34036083 | 2 | 66815719 | T | C | 0.6576 | -0.01547 | 0.006 | 2.066E-08 | 1331010 | 0.000108 | 143.51 |
| rs34967082 | 2 | 215382654 | A | G | 0.4136 | 0.01536 | 0.006 | 4.34E-09 | 1331010 | 0.000114 | 152.34 |
| rs4664299 | 2 | 160570033 | T | C | 0.2349 | -0.01773 | 0.007 | 4.953E-09 | 1331010 | 0.000113 | 150.39 |
| rs55772859 | 2 | 208042581 | A | C | 0.3106 | 0.018284 | 0.006 | 4.821E-11 | 1331010 | 0.000143 | 190.59 |
| rs56097173 | 2 | 44262449 | T | C | 0.6808 | 0.017451 | 0.006 | 2.693E-10 | 1331010 | 0.000132 | 176.19 |
| rs62149809 | 2 | 73853830 | A | G | 0.986 | 0.064083 | 0.025 | 5.713E-09 | 1331010 | 0.000113 | 150.92 |
| rs62158170 | 2 | 114082175 | A | G | 0.7856 | 0.028571 | 0.007 | 1.203E-19 | 1331010 | 0.000275 | 366.11 |
| rs62194948 | 2 | 239222376 | C | G | 0.275 | 0.017033 | 0.007 | 4.644E-09 | 1331010 | 0.000116 | 154.00 |
| rs62213452 | 2 | 210380152 | T | G | 0.2788 | 0.016197 | 0.007 | 2.385E-08 | 1331010 | 0.000106 | 140.44 |
| rs6545798 | 2 | 60521311 | A | T | 0.4104 | -0.01773 | 0.006 | 1.186E-11 | 1331010 | 0.000152 | 202.49 |
| rs6734957 | 2 | 42813247 | T | G | 0.2388 | -0.01818 | 0.007 | 1.817E-09 | 1331010 | 0.00012 | 159.97 |
| rs6756610 | 2 | 147480394 | C | G | 0.6291 | 0.016197 | 0.006 | 1.135E-09 | 1331010 | 0.000122 | 162.98 |
| rs72820274 | 2 | 104412924 | A | G | 0.417 | 0.01494 | 0.006 | 1.279E-08 | 1331010 | 0.000109 | 144.47 |
| rs75452188 | 2 | 67134426 | A | G | 0.878 | 0.022428 | 0.009 | 1.583E-08 | 1331010 | 0.000108 | 143.45 |
| rs7566062 | 2 | 66972843 | T | C | 0.2248 | 0.025715 | 0.007 | 1.369E-16 | 1331010 | 0.00023 | 306.84 |
| rs7571486 | 2 | 176473295 | A | G | 0.251 | -0.01682 | 0.007 | 1.403E-08 | 1331010 | 0.000106 | 141.68 |
| rs7599697 | 2 | 239231477 | T | C | 0.3583 | -0.01592 | 0.006 | 4.999E-09 | 1331010 | 0.000117 | 155.20 |
| rs78206187 | 2 | 67022234 | A | G | 0.9442 | -0.04096 | 0.013 | 2.955E-13 | 1331010 | 0.000177 | 235.33 |
| rs823247 | 2 | 2850540 | T | C | 0.479 | -0.01592 | 0.006 | 5.246E-10 | 1331010 | 0.000127 | 168.46 |
| rs984306 | 2 | 66817402 | T | C | 0.7547 | -0.01863 | 0.007 | 7.937E-10 | 1331010 | 0.000129 | 171.15 |
| rs10865954 | 3 | 49211989 | T | C | 0.3344 | 0.018284 | 0.006 | 1.919E-11 | 1331010 | 0.000149 | 198.11 |
| rs138014720 | 3 | 50070843 | A | T | 0.9406 | 0.030195 | 0.013 | 3.461E-08 | 1331010 | 0.000102 | 135.62 |
| rs1567084 | 3 | 71435955 | A | G | 0.4981 | 0.014521 | 0.006 | 2.136E-08 | 1331010 | 0.000105 | 140.33 |
| rs1580173 | 3 | 107955515 | A | G | 0.5608 | 0.014521 | 0.006 | 2.28E-08 | 1331010 | 0.000104 | 138.26 |
| rs17025198 | 3 | 88001713 | A | G | 0.2043 | 0.017868 | 0.007 | 2.185E-08 | 1331010 | 0.000104 | 138.17 |
| rs2216427 | 3 | 180785697 | C | G | 0.6527 | 0.01536 | 0.006 | 1.595E-08 | 1331010 | 0.000107 | 142.38 |
| rs2364921 | 3 | 158522463 | T | C | 0.4691 | -0.01457 | 0.006 | 2.129E-08 | 1331010 | 0.000106 | 140.82 |
| rs35110063 | 3 | 43066558 | A | G | 0.4266 | 0.017033 | 0.006 | 8.817E-11 | 1331010 | 0.000142 | 188.95 |
| rs3774751 | 3 | 50209053 | T | G | 0.4621 | -0.01773 | 0.006 | 7.322E-12 | 1331010 | 0.000156 | 208.00 |
| rs4260410 | 3 | 178469932 | T | C | 0.332 | 0.01494 | 0.006 | 4.869E-08 | 1331010 | 9.9E-05 | 131.79 |
| rs492858 | 3 | 155432229 | T | C | 0.0759 | -0.02872 | 0.011 | 3.458E-09 | 1331010 | 0.000116 | 154.07 |
| rs62264767 | 3 | 117642005 | A | C | 0.8531 | 0.028164 | 0.008 | 1.634E-14 | 1331010 | 0.000199 | 264.68 |
| rs6808140 | 3 | 10581380 | T | C | 0.5053 | 0.017033 | 0.006 | 5.351E-11 | 1331010 | 0.000145 | 193.09 |
| rs694786 | 3 | 173112907 | T | C | 0.4605 | -0.01909 | 0.006 | 1.969E-13 | 1331010 | 0.000181 | 241.01 |
| rs73079014 | 3 | 49863483 | T | C | 0.126 | -0.02136 | 0.009 | 3.647E-08 | 1331010 | 0.000101 | 133.80 |
| rs7432782 | 3 | 48941551 | T | C | 0.9558 | -0.03621 | 0.014 | 7.417E-09 | 1331010 | 0.000111 | 147.49 |
| rs7615602 | 3 | 18718055 | C | G | 0.2715 | -0.01728 | 0.007 | 2.589E-09 | 1331010 | 0.000118 | 157.17 |
| rs7625896 | 3 | 44062561 | A | G | 0.6545 | 0.015779 | 0.006 | 5.279E-09 | 1331010 | 0.000113 | 149.89 |
| rs11722569 | 4 | 112822731 | T | C | 0.6586 | 0.01494 | 0.006 | 2.912E-08 | 1331010 | 0.0001 | 133.62 |
| rs13135092 | 4 | 103198082 | A | G | 0.9175 | -0.03858 | 0.011 | 2.527E-16 | 1331010 | 0.000225 | 299.96 |
| rs13138995 | 4 | 148987430 | A | G | 0.3896 | 0.01494 | 0.006 | 1.974E-08 | 1331010 | 0.000106 | 141.32 |
| rs16990210 | 4 | 34720226 | T | C | 0.8478 | -0.02 | 0.008 | 1.965E-08 | 1331010 | 0.000103 | 137.37 |
| rs17005118 | 4 | 82288564 | A | G | 0.264 | 0.018284 | 0.007 | 6.128E-10 | 1331010 | 0.00013 | 172.94 |
| rs2903385 | 4 | 106094427 | A | G | 0.4844 | 0.0187 | 0.006 | 4.527E-13 | 1331010 | 0.000175 | 232.55 |
| rs4699157 | 4 | 106055212 | T | C | 0.958 | -0.03527 | 0.015 | 3.981E-08 | 1331010 | 0.0001 | 133.25 |
| rs62301574 | 4 | 22050165 | C | G | 0.7996 | -0.01818 | 0.007 | 1.37E-08 | 1331010 | 0.000106 | 141.02 |
| rs72657797 | 4 | 90820809 | T | C | 0.1759 | -0.02411 | 0.008 | 1.522E-12 | 1331010 | 0.000169 | 224.33 |
| rs12187443 | 5 | 102660400 | T | C | 0.6681 | 0.017451 | 0.006 | 1.641E-10 | 1331010 | 0.000135 | 179.78 |
| rs12520974 | 5 | 61514611 | T | C | 0.4846 | -0.01547 | 0.006 | 1.685E-09 | 1331010 | 0.00012 | 159.19 |
| rs152555 | 5 | 106849674 | A | G | 0.8544 | -0.02273 | 0.008 | 4.831E-10 | 1331010 | 0.000129 | 171.17 |
| rs16903122 | 5 | 87693561 | T | C | 0.2487 | 0.024075 | 0.007 | 9.038E-16 | 1331010 | 0.000217 | 288.35 |
| rs17083297 | 5 | 92995477 | A | C | 0.1766 | -0.01909 | 0.008 | 1.6E-08 | 1331010 | 0.000106 | 141.05 |
| rs17223714 | 5 | 50492629 | A | G | 0.7885 | 0.019947 | 0.007 | 2.435E-10 | 1331010 | 0.000133 | 176.65 |
| rs17367725 | 5 | 107112116 | T | C | 0.3513 | -0.01547 | 0.006 | 9.285E-09 | 1331010 | 0.000109 | 145.25 |
| rs2431108 | 5 | 103947968 | T | C | 0.672 | -0.02319 | 0.006 | 7.83E-17 | 1331010 | 0.000237 | 315.66 |
| rs35539975 | 5 | 91607148 | A | G | 0.7785 | 0.018284 | 0.007 | 4.492E-09 | 1331010 | 0.000115 | 153.48 |
| rs37445 | 5 | 106899684 | A | G | 0.3905 | -0.01547 | 0.006 | 4.882E-09 | 1331010 | 0.000114 | 151.70 |
| rs4502882 | 5 | 153093998 | T | C | 0.6578 | -0.01682 | 0.006 | 7.961E-10 | 1331010 | 0.000127 | 169.65 |
| rs55972276 | 5 | 135653737 | A | C | 0.1366 | 0.031812 | 0.009 | 4.19E-17 | 1331010 | 0.000239 | 317.81 |
| rs62383308 | 5 | 165460085 | A | G | 0.0805 | -0.02595 | 0.011 | 3.975E-08 | 1331010 | 9.97E-05 | 132.69 |
| rs6601080 | 5 | 179511043 | A | G | 0.6762 | 0.01536 | 0.006 | 2.211E-08 | 1331010 | 0.000103 | 137.52 |
| rs6888135 | 5 | 141254063 | A | C | 0.4965 | 0.016616 | 0.006 | 1.207E-10 | 1331010 | 0.000138 | 183.75 |
| rs701394 | 5 | 80296487 | A | G | 0.6376 | -0.01547 | 0.006 | 6.826E-09 | 1331010 | 0.000111 | 147.27 |
| rs71575448 | 5 | 106918329 | A | G | 0.8602 | 0.022016 | 0.009 | 3.38E-09 | 1331010 | 0.000117 | 155.18 |
| rs8180457 | 5 | 107209814 | T | C | 0.1573 | -0.02411 | 0.008 | 1.12E-11 | 1331010 | 0.000154 | 205.13 |
| rs10944696 | 6 | 94498850 | A | G | 0.2978 | -0.01637 | 0.007 | 7.994E-09 | 1331010 | 0.000112 | 149.26 |
| rs10947428 | 6 | 33647058 | T | C | 0.7858 | -0.02965 | 0.007 | 9.057E-21 | 1331010 | 0.000296 | 394.10 |
| rs10947690 | 6 | 37631768 | A | G | 0.7408 | -0.02045 | 0.007 | 4.039E-12 | 1331010 | 0.000161 | 213.83 |
| rs10947987 | 6 | 41754370 | T | C | 0.4431 | -0.01412 | 0.006 | 4.08E-08 | 1331010 | 9.85E-05 | 131.07 |
| rs1147852 | 6 | 147980909 | A | G | 0.3095 | 0.017033 | 0.006 | 9.935E-10 | 1331010 | 0.000124 | 165.08 |
| rs11756035 | 6 | 18843810 | C | G | 0.1281 | 0.022016 | 0.009 | 1.288E-08 | 1331010 | 0.000108 | 144.13 |
| rs138678612 | 6 | 30932223 | A | G | 0.9783 | -0.05061 | 0.02 | 1.409E-08 | 1331010 | 0.000109 | 144.76 |
| rs238869 | 6 | 29355113 | T | C | 0.6229 | -0.01457 | 0.006 | 3.356E-08 | 1331010 | 9.98E-05 | 132.82 |
| rs2388840 | 6 | 99598756 | A | G | 0.5757 | -0.01592 | 0.006 | 1.368E-09 | 1331010 | 0.000124 | 164.89 |
| rs3131638 | 6 | 31475127 | A | G | 0.2261 | -0.01909 | 0.007 | 7.88E-10 | 1331010 | 0.000128 | 169.74 |
| rs314281 | 6 | 105400605 | T | C | 0.4531 | -0.01863 | 0.006 | 6.029E-13 | 1331010 | 0.000172 | 229.10 |
| rs4709655 | 6 | 163280204 | T | C | 0.1191 | -0.02365 | 0.009 | 3.088E-09 | 1331010 | 0.000117 | 156.23 |
| rs62429521 | 6 | 140324582 | A | C | 0.1456 | 0.022016 | 0.008 | 1.776E-09 | 1331010 | 0.000121 | 160.53 |
| rs6457796 | 6 | 34828553 | T | C | 0.7313 | -0.01682 | 0.007 | 1.12E-08 | 1331010 | 0.000111 | 148.09 |
| rs728017 | 6 | 124292594 | A | G | 0.3864 | -0.01502 | 0.006 | 9.509E-09 | 1331010 | 0.000107 | 142.46 |
| rs9394502 | 6 | 38452503 | T | C | 0.3343 | -0.02365 | 0.006 | 7.756E-18 | 1331010 | 0.000249 | 331.43 |
| rs12540241 | 7 | 3824141 | A | T | 0.1926 | -0.02 | 0.008 | 1.576E-09 | 1331010 | 0.000124 | 165.55 |
| rs12666306 | 7 | 115082406 | A | G | 0.5017 | 0.018284 | 0.006 | 2.24E-12 | 1331010 | 0.000167 | 222.52 |
| rs1357685 | 7 | 109200331 | T | C | 0.4735 | 0.014521 | 0.006 | 1.385E-08 | 1331010 | 0.000105 | 139.94 |
| rs17520265 | 7 | 119674508 | A | G | 0.0342 | -0.03953 | 0.016 | 2.872E-08 | 1331010 | 0.000103 | 137.41 |
| rs190073 | 7 | 10985188 | A | G | 0.4141 | -0.01457 | 0.006 | 2.857E-08 | 1331010 | 0.000103 | 137.19 |
| rs2598293 | 7 | 133989882 | T | C | 0.4763 | 0.01536 | 0.006 | 2.478E-09 | 1331010 | 0.000118 | 156.67 |
| rs521484 | 7 | 49894349 | A | G | 0.7668 | -0.01728 | 0.007 | 1.53E-08 | 1331010 | 0.000107 | 142.10 |
| rs6465151 | 7 | 88310899 | T | C | 0.1134 | 0.024486 | 0.009 | 1.902E-09 | 1331010 | 0.000121 | 160.48 |
| rs670501 | 7 | 108625185 | T | C | 0.2133 | 0.022841 | 0.007 | 7.398E-13 | 1331010 | 0.000175 | 233.08 |
| rs6967168 | 7 | 132672192 | T | G | 0.7544 | -0.01909 | 0.007 | 1.39E-10 | 1331010 | 0.000135 | 179.73 |
| rs6973090 | 7 | 102008352 | A | G | 0.25 | -0.01637 | 0.007 | 4.312E-08 | 1331010 | 0.000101 | 133.83 |
| rs6978112 | 7 | 1966841 | T | C | 0.4105 | 0.01494 | 0.006 | 2.11E-08 | 1331010 | 0.000108 | 143.81 |
| rs75932578 | 7 | 106844694 | T | C | 0.2159 | -0.01728 | 0.007 | 4.149E-08 | 1331010 | 0.000101 | 134.52 |
| rs940780 | 7 | 3323848 | T | C | 0.3588 | 0.016616 | 0.006 | 8.498E-10 | 1331010 | 0.000127 | 169.10 |
| rs10955647 | 8 | 114154187 | T | G | 0.5321 | 0.014521 | 0.006 | 1.836E-08 | 1331010 | 0.000105 | 139.76 |
| rs17643634 | 8 | 91650818 | T | C | 0.165 | -0.02595 | 0.008 | 1.341E-13 | 1331010 | 0.000186 | 247.01 |
| rs2737240 | 8 | 116657235 | A | G | 0.7076 | 0.015779 | 0.007 | 3.373E-08 | 1331010 | 0.000103 | 137.14 |
| rs28552587 | 8 | 103356226 | A | G | 0.5641 | 0.014521 | 0.006 | 3.299E-08 | 1331010 | 0.000104 | 138.03 |
| rs28611339 | 8 | 10170037 | T | G | 0.1282 | 0.025306 | 0.009 | 8.456E-11 | 1331010 | 0.000143 | 190.55 |
| rs4588900 | 8 | 73890425 | A | G | 0.5164 | 0.014521 | 0.006 | 1.571E-08 | 1331010 | 0.000105 | 140.18 |
| rs671985 | 8 | 60914783 | A | G | 0.4516 | -0.01637 | 0.006 | 2.79E-10 | 1331010 | 0.000133 | 176.77 |
| rs871994 | 8 | 35190619 | A | C | 0.4352 | 0.01536 | 0.006 | 5.498E-09 | 1331010 | 0.000116 | 154.39 |
| rs10756571 | 9 | 14534505 | T | C | 0.6853 | 0.015779 | 0.006 | 1.799E-08 | 1331010 | 0.000107 | 142.95 |
| rs10758593 | 9 | 4292083 | A | G | 0.3989 | -0.01547 | 0.006 | 4.899E-09 | 1331010 | 0.000115 | 152.83 |
| rs10761240 | 9 | 96361922 | A | G | 0.3963 | -0.01863 | 0.006 | 2.122E-12 | 1331010 | 0.000166 | 221.19 |
| rs118166957 | 9 | 8858043 | T | C | 0.1591 | 0.029384 | 0.008 | 1.951E-16 | 1331010 | 0.000231 | 307.57 |
| rs1927902 | 9 | 120518991 | T | C | 0.2542 | 0.022841 | 0.007 | 1.147E-14 | 1331010 | 0.000198 | 263.34 |
| rs2792990 | 9 | 125621610 | C | G | 0.8553 | 0.023664 | 0.008 | 1.152E-10 | 1331010 | 0.000139 | 184.51 |
| rs4090240 | 9 | 77118987 | T | C | 0.2775 | -0.01682 | 0.007 | 8.455E-09 | 1331010 | 0.000114 | 151.10 |
| rs6597649 | 9 | 133786652 | T | C | 0.3994 | 0.014521 | 0.006 | 3.048E-08 | 1331010 | 0.000101 | 134.65 |
| rs7040224 | 9 | 134886837 | A | G | 0.3161 | 0.016197 | 0.006 | 4.242E-09 | 1331010 | 0.000113 | 151.00 |
| rs72773790 | 9 | 139109080 | T | C | 0.6732 | 0.016197 | 0.006 | 3.714E-09 | 1331010 | 0.000115 | 153.67 |
| rs77641763 | 9 | 140265782 | T | C | 0.122 | 0.031004 | 0.009 | 6.53E-15 | 1331010 | 0.000206 | 274.16 |
| rs10825503 | 10 | 57177470 | T | G | 0.4873 | 0.014521 | 0.006 | 1.426E-08 | 1331010 | 0.000105 | 140.24 |
| rs12251016 | 10 | 21821918 | A | T | 0.6559 | -0.01682 | 0.006 | 3.89E-10 | 1331010 | 0.000128 | 170.10 |
| rs224029 | 10 | 64519299 | T | C | 0.3995 | -0.01682 | 0.006 | 2.507E-10 | 1331010 | 0.000136 | 180.80 |
| rs7475916 | 10 | 77771194 | C | G | 0.3533 | -0.01592 | 0.006 | 6.7E-09 | 1331010 | 0.000116 | 154.23 |
| rs10898940 | 11 | 73455292 | A | C | 0.5173 | 0.01494 | 0.006 | 8.093E-09 | 1331010 | 0.000111 | 148.39 |
| rs11605348 | 11 | 47606483 | A | G | 0.3495 | -0.01954 | 0.006 | 7.011E-13 | 1331010 | 0.000174 | 231.17 |
| rs12790660 | 11 | 57667222 | T | C | 0.6844 | -0.01728 | 0.006 | 4.486E-10 | 1331010 | 0.000129 | 171.64 |
| rs214934 | 11 | 17193475 | A | T | 0.3123 | -0.01637 | 0.006 | 3.163E-09 | 1331010 | 0.000115 | 153.29 |
| rs4592425 | 11 | 62697813 | T | G | 0.6965 | 0.017451 | 0.006 | 4.31E-10 | 1331010 | 0.000129 | 171.39 |
| rs524859 | 11 | 66041079 | A | G | 0.3601 | -0.01909 | 0.006 | 1.478E-12 | 1331010 | 0.000168 | 223.53 |
| rs56133505 | 11 | 72348039 | A | G | 0.5369 | 0.017868 | 0.006 | 5.591E-12 | 1331010 | 0.000159 | 211.34 |
| rs566673 | 11 | 66401373 | T | G | 0.5351 | -0.01682 | 0.006 | 1.18E-10 | 1331010 | 0.000141 | 187.49 |
| rs647905 | 11 | 121534938 | T | C | 0.5409 | 0.014521 | 0.006 | 2.872E-08 | 1331010 | 0.000105 | 139.39 |
| rs6589988 | 11 | 99126016 | A | G | 0.6757 | -0.01637 | 0.006 | 4.699E-09 | 1331010 | 0.000117 | 156.41 |
| rs667730 | 11 | 83277325 | T | C | 0.5788 | 0.014521 | 0.006 | 2.263E-08 | 1331010 | 0.000103 | 136.85 |
| rs72899452 | 11 | 45415577 | T | C | 0.0648 | 0.032216 | 0.012 | 1.002E-09 | 1331010 | 0.000126 | 167.45 |
| rs79693059 | 11 | 72340686 | C | G | 0.9158 | -0.03152 | 0.011 | 1.607E-11 | 1331010 | 0.000153 | 203.93 |
| rs1167132 | 12 | 43484487 | T | C | 0.3917 | 0.01536 | 0.006 | 8.732E-09 | 1331010 | 0.000112 | 149.66 |
| rs12310246 | 12 | 84700945 | A | G | 0.2489 | 0.019532 | 0.007 | 4.744E-11 | 1331010 | 0.000143 | 189.88 |
| rs28582096 | 12 | 123856998 | A | G | 0.205 | -0.02365 | 0.007 | 1.738E-13 | 1331010 | 0.000182 | 242.70 |
| rs324017 | 12 | 57487814 | A | C | 0.294 | 0.017033 | 0.007 | 1.609E-09 | 1331010 | 0.00012 | 160.33 |
| rs4767645 | 12 | 118385788 | T | G | 0.4614 | -0.01592 | 0.006 | 6.47E-10 | 1331010 | 0.000126 | 167.75 |
| rs61921611 | 12 | 66367726 | T | C | 0.692 | -0.01909 | 0.006 | 7.839E-12 | 1331010 | 0.000155 | 206.76 |
| rs6606731 | 12 | 109982578 | A | T | 0.1923 | 0.0187 | 0.008 | 1.506E-08 | 1331010 | 0.000109 | 144.61 |
| rs7486418 | 12 | 84336911 | T | G | 0.657 | 0.017868 | 0.006 | 6.837E-11 | 1331010 | 0.000144 | 191.55 |
| rs1031654 | 13 | 54382035 | A | C | 0.7996 | -0.02228 | 0.007 | 3.881E-12 | 1331010 | 0.000159 | 211.71 |
| rs11149313 | 13 | 85294881 | A | G | 0.7297 | 0.017451 | 0.007 | 2.376E-09 | 1331010 | 0.00012 | 159.91 |
| rs117630493 | 13 | 54018867 | C | G | 0.9726 | -0.04383 | 0.018 | 3.608E-08 | 1331010 | 0.000102 | 136.31 |
| rs11838830 | 13 | 60362013 | A | G | 0.9436 | -0.0348 | 0.013 | 5.196E-10 | 1331010 | 0.000129 | 171.57 |
| rs1536053 | 13 | 111982291 | T | C | 0.3157 | -0.01637 | 0.006 | 6.043E-09 | 1331010 | 0.000116 | 154.20 |
| rs2389631 | 13 | 96932868 | A | C | 0.6666 | -0.01728 | 0.006 | 2.027E-10 | 1331010 | 0.000133 | 176.61 |
| rs2491124 | 13 | 53784083 | T | C | 0.5758 | 0.021189 | 0.006 | 8.809E-16 | 1331010 | 0.000219 | 292.00 |
| rs6562066 | 13 | 60532796 | T | C | 0.3688 | 0.017033 | 0.006 | 1.379E-10 | 1331010 | 0.000135 | 179.82 |
| rs79204944 | 13 | 53969796 | A | G | 0.0449 | 0.034227 | 0.014 | 4.244E-08 | 1331010 | 0.0001 | 133.75 |
| rs7992992 | 13 | 54721699 | A | G | 0.1286 | 0.022016 | 0.009 | 1.152E-08 | 1331010 | 0.000109 | 144.60 |
| rs8181889 | 13 | 53613990 | A | G | 0.3992 | -0.01637 | 0.006 | 8.898E-10 | 1331010 | 0.000129 | 171.19 |
| rs9316619 | 13 | 53978628 | T | C | 0.8249 | 0.019947 | 0.008 | 5.503E-09 | 1331010 | 0.000115 | 153.00 |
| rs9527083 | 13 | 53991125 | A | G | 0.6705 | -0.03292 | 0.006 | 1.611E-32 | 1331010 | 0.000479 | 637.68 |
| rs9563886 | 13 | 61720066 | T | C | 0.6063 | -0.01457 | 0.006 | 3.078E-08 | 1331010 | 0.000101 | 134.97 |
| rs4981170 | 14 | 33412996 | A | G | 0.1943 | -0.02365 | 0.008 | 7.331E-13 | 1331010 | 0.000175 | 233.13 |
| rs1038093 | 15 | 74012409 | T | C | 0.6282 | 0.017033 | 0.006 | 2.466E-10 | 1331010 | 0.000136 | 180.42 |
| rs12912299 | 15 | 38897857 | T | C | 0.4893 | -0.01863 | 0.006 | 4.416E-13 | 1331010 | 0.000174 | 231.03 |
| rs12917449 | 15 | 74331659 | A | C | 0.8061 | -0.01818 | 0.008 | 2.965E-08 | 1331010 | 0.000103 | 137.56 |
| rs176644 | 15 | 89913632 | T | G | 0.4036 | 0.01536 | 0.006 | 9.491E-09 | 1331010 | 0.000114 | 151.19 |
| rs4702 | 15 | 91426560 | A | G | 0.5562 | -0.02091 | 0.006 | 6.777E-16 | 1331010 | 0.000216 | 287.28 |
| rs715338 | 15 | 57215867 | A | G | 0.5776 | 0.017868 | 0.006 | 7.853E-12 | 1331010 | 0.000156 | 207.38 |
| rs7168238 | 15 | 66709386 | C | G | 0.0743 | 0.027757 | 0.011 | 1.798E-08 | 1331010 | 0.000106 | 141.08 |
| rs7402939 | 15 | 99183876 | T | C | 0.376 | -0.01547 | 0.006 | 5.191E-09 | 1331010 | 0.000112 | 149.54 |
| rs1015438 | 16 | 51177517 | A | G | 0.1882 | 0.025306 | 0.008 | 2.509E-14 | 1331010 | 0.000196 | 260.50 |
| rs12924275 | 16 | 9191790 | T | C | 0.268 | 0.016616 | 0.007 | 1.925E-08 | 1331010 | 0.000108 | 144.19 |
| rs2398144 | 16 | 56352854 | A | C | 0.3947 | 0.016616 | 0.006 | 5.091E-10 | 1331010 | 0.000132 | 175.60 |
| rs3184470 | 16 | 715164 | A | G | 0.3507 | -0.01637 | 0.006 | 9.726E-10 | 1331010 | 0.000122 | 162.53 |
| rs34214423 | 16 | 52303107 | A | C | 0.8087 | 0.019532 | 0.008 | 3.178E-09 | 1331010 | 0.000118 | 157.12 |
| rs35322724 | 16 | 77137324 | A | C | 0.5774 | 0.021189 | 0.006 | 3.753E-16 | 1331010 | 0.000219 | 291.71 |
| rs3902952 | 16 | 61647589 | T | C | 0.1881 | 0.020775 | 0.008 | 2.545E-10 | 1331010 | 0.000132 | 175.49 |
| rs4238755 | 16 | 52746089 | A | C | 0.2638 | -0.01863 | 0.007 | 2.299E-10 | 1331010 | 0.000135 | 179.55 |
| rs45453598 | 16 | 52637892 | A | T | 0.169 | 0.020361 | 0.008 | 4.422E-09 | 1331010 | 0.000116 | 155.01 |
| rs4788203 | 16 | 29978827 | A | G | 0.4334 | -0.01502 | 0.006 | 6.323E-09 | 1331010 | 0.000111 | 147.55 |
| rs66674044 | 16 | 19904344 | A | T | 0.8573 | -0.02595 | 0.009 | 2.184E-12 | 1331010 | 0.000165 | 219.32 |
| rs67501351 | 16 | 20006745 | C | G | 0.7451 | 0.019532 | 0.007 | 5.363E-11 | 1331010 | 0.000145 | 192.90 |
| rs9931543 | 16 | 56128782 | T | C | 0.7359 | 0.020775 | 0.007 | 1.111E-12 | 1331010 | 0.000168 | 223.34 |
| rs11650304 | 17 | 46035001 | C | G | 0.9308 | 0.028978 | 0.012 | 1.227E-08 | 1331010 | 0.000108 | 144.00 |
| rs1553754 | 17 | 46563707 | T | G | 0.5621 | -0.01457 | 0.006 | 3.507E-08 | 1331010 | 0.000105 | 139.18 |
| rs2447094 | 17 | 2294048 | A | C | 0.4687 | -0.01457 | 0.006 | 2.503E-08 | 1331010 | 0.000106 | 140.81 |
| rs34490907 | 17 | 26933741 | C | G | 0.8878 | 0.023252 | 0.009 | 1.755E-08 | 1331010 | 0.000108 | 143.38 |
| rs4643373 | 17 | 47123423 | T | C | 0.7006 | 0.017868 | 0.007 | 1.582E-10 | 1331010 | 0.000134 | 178.29 |
| rs4790076 | 17 | 2243628 | T | C | 0.1742 | 0.020775 | 0.008 | 1.757E-09 | 1331010 | 0.000124 | 165.31 |
| rs62068188 | 17 | 2400876 | T | C | 0.8339 | 0.021189 | 0.008 | 1.184E-09 | 1331010 | 0.000124 | 165.57 |
| rs7214267 | 17 | 43157709 | A | G | 0.581 | -0.01909 | 0.006 | 5.093E-13 | 1331010 | 0.000177 | 236.16 |
| rs8076183 | 17 | 61024696 | T | C | 0.4485 | -0.01637 | 0.006 | 2.754E-10 | 1331010 | 0.000133 | 176.55 |
| rs9889282 | 17 | 50259142 | A | C | 0.6129 | -0.01818 | 0.006 | 4.697E-12 | 1331010 | 0.000157 | 208.81 |
| rs10502966 | 18 | 50748499 | A | G | 0.582 | -0.01682 | 0.006 | 8.537E-11 | 1331010 | 0.000138 | 183.35 |
| rs12605642 | 18 | 31313965 | T | G | 0.4864 | 0.01536 | 0.006 | 2.129E-09 | 1331010 | 0.000118 | 156.91 |
| rs60565673 | 18 | 52906830 | T | G | 0.6211 | -0.01863 | 0.006 | 1.591E-12 | 1331010 | 0.000163 | 217.57 |
| rs9964420 | 18 | 56824041 | A | C | 0.301 | 0.01536 | 0.007 | 4.54E-08 | 1331010 | 9.93E-05 | 132.15 |
| rs12983032 | 19 | 5073447 | A | G | 0.3434 | -0.01863 | 0.006 | 1.065E-11 | 1331010 | 0.000157 | 208.46 |
| rs429358 | 19 | 45411941 | T | C | 0.8458 | 0.019947 | 0.008 | 2.125E-08 | 1331010 | 0.000104 | 138.15 |
| rs6510033 | 19 | 30710785 | A | G | 0.7253 | -0.01592 | 0.007 | 4.661E-08 | 1331010 | 0.000101 | 134.49 |
| rs908668 | 19 | 56134038 | T | C | 0.2081 | 0.021603 | 0.007 | 1.411E-11 | 1331010 | 0.000154 | 204.76 |
| rs2867690 | 20 | 41972028 | T | C | 0.1819 | 0.018284 | 0.008 | 3.701E-08 | 1331010 | 9.95E-05 | 132.45 |
| rs6019663 | 20 | 47774512 | T | C | 0.293 | 0.017451 | 0.007 | 6.474E-10 | 1331010 | 0.000126 | 167.95 |
| rs742760 | 20 | 50985290 | A | T | 0.8155 | 0.0187 | 0.008 | 2.475E-08 | 1331010 | 0.000105 | 140.08 |
| rs76145129 | 20 | 62670427 | T | G | 0.1238 | -0.02182 | 0.009 | 2.733E-08 | 1331010 | 0.000103 | 137.49 |
| rs910187 | 20 | 45841052 | A | G | 0.373 | -0.01502 | 0.006 | 1.631E-08 | 1331010 | 0.000106 | 140.52 |
| rs2838787 | 21 | 46539725 | A | G | 0.3924 | -0.01547 | 0.006 | 7.654E-09 | 1331010 | 0.000114 | 151.96 |
| rs11090039 | 22 | 41496800 | A | G | 0.2871 | 0.017033 | 0.007 | 1.822E-09 | 1331010 | 0.000119 | 158.10 |
| rs17324524 | X | 133134421 | T | C | 0.883 | -0.02503 | 0.009 | 5.01E-10 | 1331010 | 0.000129 | 172.29 |
| rs62590551 | X | 21494390 | A | G | 0.9029 | 0.023664 | 0.01 | 4.61E-09 | 1331010 | 9.82E-05 | 130.70 |

SNP: single nucleotide polymorphism; EA: effect allele; OA: other allele; SE: standard error; R2: proportion of phenotypic variance explained by SNPs.

**Supplementary Table 7. Main characteristics of SNPs to instrument sleep duration.**

| **SNP** | **Chr** | **Position** | **EA** | **OA** | **EAF** | **Beta** | **SE** | **P value** | **Sample size** | **R^2^** | **F** |
| --- | --- | --- | --- | --- | --- | --- | --- | --- | --- | --- | --- |
| rs12567114 | 1 | 98527951 | A | G | 0.276 | 0.89 | 0.152418 | 4.3E-09 | 446118 | 7.64E-05 | 34.10 |
| rs61796569 | 1 | 66476437 | T | C | 0.27 | 0.927 | 0.153866 | 1.5E-09 | 446118 | 8.14E-05 | 36.30 |
| rs915416 | 1 | 34731984 | C | G | 0.29 | 1.156 | 0.149671 | 9.9E-15 | 446118 | 0.000134 | 59.65 |
| rs62120041 | 2 | 9185564 | T | C | 0.934 | 1.567 | 0.274498 | 9.6E-09 | 446118 | 7.3E-05 | 32.59 |
| rs72804080 | 2 | 59358659 | G | A | 0.15 | 1.068 | 0.191785 | 2.9E-08 | 446118 | 6.95E-05 | 31.01 |
| rs374153 | 2 | 40382712 | C | T | 0.158 | 1.057 | 0.186185 | 9.1E-09 | 446118 | 7.22E-05 | 32.23 |
| rs4538155 | 2 | 1.57E+08 | T | C | 0.647 | 0.779 | 0.14243 | 3.6E-08 | 446118 | 6.7E-05 | 29.91 |
| rs11885663 | 2 | 1.67E+08 | T | C | 0.248 | 0.973 | 0.157087 | 8.6E-10 | 446118 | 8.6E-05 | 38.37 |
| rs10173260 | 2 | 2.1E+08 | C | T | 0.606 | 0.77 | 0.138793 | 2.9E-08 | 446118 | 6.9E-05 | 30.78 |
| rs12611523 | 2 | 1.39E+08 | A | G | 0.545 | 0.758 | 0.136576 | 3.1E-08 | 446118 | 6.9E-05 | 30.80 |
| rs4128364 | 2 | 1.48E+08 | C | T | 0.339 | 0.876 | 0.143452 | 1.4E-09 | 446118 | 8.36E-05 | 37.29 |
| rs7556815 | 2 | 1.14E+08 | A | G | 0.219 | 2.443 | 0.164414 | 1.3E-49 | 446118 | 0.000495 | 220.78 |
| rs112230981 | 3 | 55879269 | A | G | 0.95 | 1.892 | 0.313672 | 2.2E-09 | 446118 | 8.15E-05 | 36.38 |
| rs7644809 | 3 | 1.08E+08 | T | C | 0.422 | 0.784 | 0.138089 | 1.6E-08 | 446118 | 7.22E-05 | 32.23 |
| rs7616632 | 3 | 1.37E+08 | T | G | 0.522 | 0.792 | 0.136153 | 4.3E-09 | 446118 | 7.58E-05 | 33.84 |
| rs13088093 | 3 | 1.36E+08 | G | T | 0.336 | 0.976 | 0.144141 | 7E-12 | 446118 | 0.000103 | 45.85 |
| rs13109404 | 4 | 1.03E+08 | T | G | 0.928 | 1.872 | 0.264497 | 1.4E-12 | 446118 | 0.000112 | 50.09 |
| rs17427571 | 4 | 82254908 | A | G | 0.684 | 0.83 | 0.146126 | 1.3E-08 | 446118 | 7.23E-05 | 32.26 |
| rs35531607 | 4 | 92533225 | C | T | 0.474 | 0.77 | 0.136367 | 1.5E-08 | 446118 | 7.15E-05 | 31.88 |
| rs2192528 | 4 | 18327896 | A | G | 0.48 | 0.802 | 0.136152 | 2.7E-09 | 446118 | 7.78E-05 | 34.70 |
| rs460692 | 5 | 3126584 | C | T | 0.137 | 1.263 | 0.199898 | 3.6E-10 | 446118 | 8.95E-05 | 39.92 |
| rs151014368 | 5 | 1.77E+08 | A | G | 0.206 | 0.966 | 0.169175 | 9.1E-09 | 446118 | 7.31E-05 | 32.60 |
| rs180769 | 5 | 1.36E+08 | T | C | 0.425 | 0.763 | 0.13763 | 2.3E-08 | 446118 | 6.89E-05 | 30.73 |
| rs11567976 | 5 | 1.38E+08 | T | C | 0.571 | 0.768 | 0.13711 | 2.1E-08 | 446118 | 7.03E-05 | 31.38 |
| rs365663 | 5 | 1428883 | A | G | 0.546 | 0.878 | 0.136714 | 1E-10 | 446118 | 9.24E-05 | 41.24 |
| rs56372231 | 5 | 1.02E+08 | T | C | 0.334 | 1.017 | 0.143989 | 2.2E-12 | 446118 | 0.000112 | 49.89 |
| rs80193650 | 6 | 33464363 | G | A | 0.162 | 1.01 | 0.184034 | 4.1E-08 | 446118 | 6.75E-05 | 30.12 |
| rs2231265 | 6 | 89790201 | G | A | 0.772 | 0.897 | 0.16195 | 2.7E-08 | 446118 | 6.88E-05 | 30.68 |
| rs113113059 | 6 | 43160375 | T | C | 0.78 | 0.968 | 0.164239 | 8.4E-09 | 446118 | 7.79E-05 | 34.74 |
| rs9345234 | 6 | 93162639 | C | A | 0.578 | 0.781 | 0.137936 | 1.8E-08 | 446118 | 7.19E-05 | 32.06 |
| rs9382445 | 6 | 54937974 | T | C | 0.623 | 0.872 | 0.140032 | 4.8E-10 | 446118 | 8.69E-05 | 38.78 |
| rs7806045 | 7 | 1.33E+08 | T | C | 0.755 | 0.887 | 0.157546 | 1.4E-08 | 446118 | 7.1E-05 | 31.70 |
| rs2079070 | 7 | 1.14E+08 | C | G | 0.265 | 1.053 | 0.153983 | 7.5E-12 | 446118 | 0.000105 | 46.76 |
| rs73219758 | 8 | 14279446 | G | A | 0.708 | 0.984 | 0.149716 | 5.6E-11 | 446118 | 9.68E-05 | 43.20 |
| rs330088 | 8 | 9149746 | C | T | 0.547 | 0.868 | 0.136607 | 2.7E-10 | 446118 | 9.05E-05 | 40.37 |
| rs1776776 | 9 | 1.4E+08 | T | C | 0.874 | 1.198 | 0.204643 | 4.9E-09 | 446118 | 7.68E-05 | 34.27 |
| rs10973207 | 9 | 37100525 | T | G | 0.158 | 1.226 | 0.187436 | 6E-11 | 446118 | 9.59E-05 | 42.78 |
| rs7915425 | 10 | 1.25E+08 | T | C | 0.175 | 1.144 | 0.179375 | 2E-10 | 446118 | 9.12E-05 | 40.67 |
| rs10761674 | 10 | 64618340 | C | T | 0.477 | 0.74 | 0.135955 | 4.2E-08 | 446118 | 6.64E-05 | 29.63 |
| rs12246842 | 10 | 21830580 | A | G | 0.46 | 0.804 | 0.136455 | 3.9E-09 | 446118 | 7.78E-05 | 34.72 |
| rs7951019 | 11 | 1.18E+08 | G | T | 0.032 | 2.213 | 0.391251 | 1.2E-08 | 446118 | 7.17E-05 | 31.99 |
| rs12791153 | 11 | 80685181 | T | A | 0.081 | 1.413 | 0.253014 | 1.9E-08 | 446118 | 6.99E-05 | 31.19 |
| rs7115226 | 11 | 1.13E+08 | A | C | 0.074 | 1.594 | 0.261479 | 1.7E-09 | 446118 | 8.33E-05 | 37.16 |
| rs1939455 | 11 | 1.02E+08 | G | T | 0.879 | 1.226 | 0.213671 | 1.2E-08 | 446118 | 7.38E-05 | 32.92 |
| rs1057703 | 11 | 1.23E+08 | G | T | 0.147 | 1.164 | 0.192397 | 1.1E-09 | 446118 | 8.2E-05 | 36.60 |
| rs11602180 | 11 | 48162453 | C | T | 0.837 | 1.095 | 0.183948 | 2.3E-09 | 446118 | 7.94E-05 | 35.44 |
| rs1553132 | 11 | 88297740 | G | A | 0.258 | 0.87 | 0.155068 | 2.5E-08 | 446118 | 7.06E-05 | 31.48 |
| rs174560 | 11 | 61581764 | C | T | 0.314 | 0.815 | 0.146205 | 2.8E-08 | 446118 | 6.96E-05 | 31.07 |
| rs1263056 | 11 | 1.17E+08 | A | G | 0.519 | 0.768 | 0.136633 | 2E-08 | 446118 | 7.08E-05 | 31.59 |
| rs1517572 | 11 | 28829882 | C | A | 0.581 | 0.879 | 0.13768 | 1.5E-10 | 446118 | 9.14E-05 | 40.76 |
| rs4592416 | 11 | 43800474 | G | A | 0.464 | 0.881 | 0.136206 | 9.3E-11 | 446118 | 9.38E-05 | 41.84 |
| rs11614986 | 12 | 1.1E+08 | A | G | 0.821 | 0.983 | 0.177061 | 2.7E-08 | 446118 | 6.91E-05 | 30.82 |
| rs34354917 | 12 | 38764559 | C | A | 0.71 | 0.825 | 0.150049 | 3.9E-08 | 446118 | 6.78E-05 | 30.23 |
| rs4767550 | 12 | 1.18E+08 | G | A | 0.414 | 0.858 | 0.138576 | 6.3E-10 | 446118 | 8.59E-05 | 38.34 |
| rs11621908 | 14 | 78495761 | C | T | 0.917 | 1.446 | 0.249779 | 5.6E-09 | 446118 | 7.51E-05 | 33.51 |
| rs61985058 | 14 | 60233841 | T | C | 0.143 | 1.116 | 0.193736 | 1.3E-08 | 446118 | 7.44E-05 | 33.18 |
| rs10483350 | 14 | 29816155 | G | A | 0.195 | 1.042 | 0.172056 | 1.5E-09 | 446118 | 8.22E-05 | 36.68 |
| rs6575005 | 14 | 26954078 | T | C | 0.758 | 0.934 | 0.158503 | 4.4E-09 | 446118 | 7.78E-05 | 34.72 |
| rs55658675 | 14 | 65554638 | C | T | 0.645 | 0.788 | 0.142135 | 2E-08 | 446118 | 6.89E-05 | 30.74 |
| rs8038326 | 15 | 47989799 | A | G | 0.727 | 0.955 | 0.152442 | 2.8E-10 | 446118 | 8.8E-05 | 39.25 |
| rs11643715 | 16 | 23909538 | G | C | 0.291 | 0.834 | 0.149795 | 3.2E-08 | 446118 | 6.95E-05 | 31.00 |
| rs3095508 | 16 | 6550400 | C | A | 0.594 | 0.921 | 0.138262 | 3.1E-11 | 446118 | 9.95E-05 | 44.37 |
| rs8050478 | 16 | 56120461 | G | A | 0.5 | 0.96 | 0.135886 | 1.7E-12 | 446118 | 0.000112 | 49.91 |
| rs7503199 | 17 | 8134275 | C | T | 0.734 | 0.885 | 0.15383 | 1E-08 | 446118 | 7.42E-05 | 33.10 |
| rs1991556 | 17 | 44083402 | G | A | 0.774 | 0.994 | 0.163424 | 1E-09 | 446118 | 8.29E-05 | 36.99 |
| rs2139261 | 17 | 21313223 | G | C | 0.749 | 1.122 | 0.173869 | 8.5E-11 | 446118 | 9.33E-05 | 41.64 |
| rs9903973 | 17 | 50571227 | C | T | 0.467 | 0.766 | 0.136316 | 2.6E-08 | 446118 | 7.08E-05 | 31.58 |
| rs205024 | 17 | 11227352 | T | C | 0.384 | 0.83 | 0.139627 | 3.9E-09 | 446118 | 7.92E-05 | 35.34 |
| rs12607679 | 18 | 53059748 | T | C | 0.738 | 1.208 | 0.15557 | 8.3E-15 | 446118 | 0.000135 | 60.29 |
| rs2072727 | 20 | 43538733 | T | C | 0.436 | 0.795 | 0.137104 | 7.9E-09 | 446118 | 7.54E-05 | 33.62 |

SNP: single nucleotide polymorphism; EA: effect allele; OA: other allele; SE: standard error; R2: proportion of phenotypic variance explained by SNPs.

**Supplementary Table 8. Main characteristics of SNPs to instrument short sleep duration.**

| **SNP** | **Chr** | **Position** | **EA** | **OA** | **EAF** | **Beta** | **SE** | **P value** | **Sample size** | **R^2^** | **F** |
| --- | --- | --- | --- | --- | --- | --- | --- | --- | --- | --- | --- |
| rs12567114 | 1 | 98527951 | G | A | 0.725 | 0.0363 | 0.0066 | 4.1E-09 | 411934 | 0.000525 | 216.55 |
| rs2820313 | 1 | 201870221 | G | A | 0.341 | 0.0305 | 0.0061 | 2.3E-09 | 411934 | 0.000418 | 172.30 |
| rs7524118 | 1 | 34736052 | C | T | 0.708 | 0.0296 | 0.0066 | 4.9E-08 | 411934 | 0.000362 | 149.28 |
| rs1380703 | 2 | 57941287 | G | A | 0.384 | 0.0354 | 0.0061 | 1.6E-11 | 411934 | 0.000593 | 244.36 |
| rs2863957 | 2 | 114089551 | C | A | 0.782 | 0.0545 | 0.0077 | 2.6E-18 | 411934 | 0.001013 | 417.59 |
| rs2014830 | 3 | 50172397 | C | T | 0.698 | 0.0296 | 0.0066 | 2.7E-08 | 411934 | 0.000369 | 152.22 |
| rs13107325 | 4 | 103188709 | T | C | 0.075 | 0.0751 | 0.0117 | 2.5E-13 | 411934 | 0.000783 | 322.61 |
| rs17005118 | 4 | 82288564 | A | G | 0.265 | 0.0296 | 0.0066 | 2.5E-09 | 411934 | 0.000341 | 140.64 |
| rs12518468 | 5 | 7249696 | C | T | 0.328 | 0.0315 | 0.0066 | 8.5E-09 | 411934 | 0.000437 | 180.26 |
| rs3776864 | 5 | 102327868 | A | C | 0.667 | 0.0315 | 0.0066 | 1.7E-08 | 411934 | 0.000441 | 181.65 |
| rs4585442 | 5 | 135508381 | G | A | 0.311 | 0.0305 | 0.0061 | 8.1E-10 | 411934 | 0.000399 | 164.29 |
| rs12661667 | 6 | 41792545 | T | C | 0.263 | 0.0276 | 0.0066 | 2.8E-08 | 411934 | 0.000295 | 121.68 |
| rs142180737 | 6 | 28344731 | C | T | 0.009 | 0.1544 | 0.0357 | 4.4E-09 | 411934 | 0.000425 | 175.25 |
| rs9321171 | 6 | 129848635 | C | T | 0.54 | 0.0315 | 0.0061 | 4.2E-08 | 411934 | 0.000493 | 203.16 |
| rs11763750 | 7 | 2080114 | G | A | 0.814 | 0.0354 | 0.0082 | 5.1E-09 | 411934 | 0.000379 | 156.37 |
| rs1229762 | 7 | 114218582 | T | C | 0.665 | 0.0373 | 0.0066 | 1.1E-12 | 411934 | 0.00062 | 255.51 |
| rs60882754 | 8 | 52886619 | A | T | 0.939 | 0.0554 | 0.0128 | 1.8E-08 | 411934 | 0.000352 | 144.88 |
| rs1607227 | 11 | 28808617 | G | T | 0.705 | 0.0305 | 0.0066 | 1.5E-09 | 411934 | 0.000387 | 159.45 |
| rs7939345 | 11 | 47980568 | T | G | 0.208 | 0.0354 | 0.0077 | 4E-08 | 411934 | 0.000413 | 170.15 |
| rs17388803 | 15 | 48027204 | C | A | 0.106 | 0.0526 | 0.0102 | 6.5E-10 | 411934 | 0.000524 | 216.12 |
| rs59779556 | 16 | 56227965 | T | G | 0.554 | 0.0247 | 0.0061 | 2E-08 | 411934 | 0.000301 | 124.23 |
| rs205024 | 17 | 11227352 | C | T | 0.617 | 0.0305 | 0.0066 | 2.7E-08 | 411934 | 0.00044 | 181.19 |
| rs12963463 | 18 | 53099093 | C | T | 0.299 | 0.0286 | 0.0066 | 1.9E-11 | 411934 | 0.000343 | 141.29 |
| rs5757675 | 22 | 39838892 | G | T | 0.26 | 0.0344 | 0.0071 | 2.7E-09 | 411934 | 0.000455 | 187.66 |

SNP: single nucleotide polymorphism; EA: effect allele; OA: other allele; SE: standard error; R2: proportion of phenotypic variance explained by SNPs.

**Supplementary Table 9. Main characteristics of SNPs to instrument time spent using computer.**

| **SNP** | **Chr** | **Position** | **EA** | **OA** | **EAF** | **Beta** | **SE** | **P value** | **Sample size** | **R^2^** | **F** |
| --- | --- | --- | --- | --- | --- | --- | --- | --- | --- | --- | --- |
| rs117405403 | 1 | 197744098 | C | G | 0.584606 | 0.011624 | 0.001848 | 3.2E-10 | 360895 | 6.56209E-05 | 23.68 |
| rs12128707 | 1 | 72588119 | G | A | 0.263894 | 0.012036 | 0.002079 | 7.1E-09 | 360895 | 5.62813E-05 | 20.31 |
| rs12145677 | 1 | 110023610 | A | G | 0.297237 | 0.015578 | 0.001999 | 6.4E-15 | 360895 | 0.000101388 | 36.59 |
| rs2120461 | 1 | 8447722 | T | C | 0.661091 | 0.012184 | 0.001926 | 2.5E-10 | 360895 | 6.65247E-05 | 24.01 |
| rs2748985 | 1 | 1853184 | C | T | 0.544439 | 0.012497 | 0.001834 | 9.399E-12 | 360895 | 7.74707E-05 | 27.96 |
| rs2761438 | 1 | 110752139 | G | A | 0.623567 | -0.01133 | 0.001882 | 1.7E-09 | 360895 | 6.03016E-05 | 21.76 |
| rs7526112 | 1 | 93747683 | G | T | 0.361752 | -0.01085 | 0.001898 | 1.1E-08 | 360895 | 5.43393E-05 | 19.61 |
| rs10208088 | 2 | 221055873 | T | C | 0.579637 | -0.01024 | 0.001849 | 3E-08 | 360895 | 5.11157E-05 | 18.45 |
| rs1037091 | 2 | 155652357 | T | C | 0.327191 | -0.01548 | 0.001983 | 5.901E-15 | 360895 | 0.000105492 | 38.08 |
| rs112600282 | 2 | 156895797 | G | A | 0.110458 | -0.01691 | 0.002923 | 7.2E-09 | 360895 | 5.62041E-05 | 20.28 |
| rs13422733 | 2 | 102010245 | T | C | 0.125533 | -0.01515 | 0.002761 | 4.1E-08 | 360895 | 5.04001E-05 | 18.19 |
| rs2032780 | 2 | 215073935 | C | T | 0.405991 | 0.01235 | 0.001875 | 4.5E-11 | 360895 | 7.3563E-05 | 26.55 |
| rs2041687 | 2 | 60405620 | G | T | 0.549172 | 0.011709 | 0.001869 | 3.8E-10 | 360895 | 6.7892E-05 | 24.50 |
| rs4852252 | 2 | 71539301 | C | T | 0.563856 | 0.010193 | 0.001839 | 3E-08 | 360895 | 5.11003E-05 | 18.44 |
| rs75550998 | 2 | 146486095 | T | G | 0.053578 | -0.02239 | 0.004095 | 4.6E-08 | 360895 | 5.08473E-05 | 18.35 |
| rs7564844 | 2 | 215335556 | A | G | 0.700653 | -0.01234 | 0.001991 | 5.6E-10 | 360895 | 6.39113E-05 | 23.07 |
| rs76112266 | 2 | 201087157 | G | C | 0.166004 | -0.01626 | 0.002455 | 3.5E-11 | 360895 | 7.31856E-05 | 26.41 |
| rs6780848 | 3 | 8179920 | G | T | 0.269946 | 0.011368 | 0.002057 | 3.3E-08 | 360895 | 5.09348E-05 | 18.38 |
| rs7630869 | 3 | 49522543 | T | C | 0.304231 | 0.016113 | 0.00198 | 4E-16 | 360895 | 0.000109919 | 39.67 |
| rs10518019 | 4 | 67959875 | G | A | 0.476252 | 0.009944 | 0.001823 | 4.9E-08 | 360895 | 4.93253E-05 | 17.80 |
| rs11942953 | 4 | 163753973 | C | T | 0.536706 | -0.01046 | 0.001839 | 1.3E-08 | 360895 | 5.43694E-05 | 19.62 |
| rs1229984 | 4 | 100239319 | C | T | 0.97263 | -0.03445 | 0.005522 | 4.4E-10 | 360895 | 6.3171E-05 | 22.80 |
| rs1395020 | 4 | 139690326 | A | G | 0.302632 | -0.011 | 0.001993 | 3.4E-08 | 360895 | 5.10675E-05 | 18.43 |
| rs2068625 | 4 | 159856739 | C | T | 0.698468 | 0.014175 | 0.001989 | 1E-12 | 360895 | 8.4635E-05 | 30.55 |
| rs2588543 | 4 | 37000406 | T | C | 0.67203 | 0.010908 | 0.00195 | 2.2E-08 | 360895 | 5.24526E-05 | 18.93 |
| rs28710456 | 4 | 152667171 | C | T | 0.49014 | -0.01041 | 0.001827 | 1.2E-08 | 360895 | 5.41182E-05 | 19.53 |
| rs79720045 | 4 | 39797668 | C | T | 0.400744 | -0.01276 | 0.001898 | 1.8E-11 | 360895 | 7.82301E-05 | 28.23 |
| rs11749912 | 5 | 88065628 | G | A | 0.574515 | -0.01288 | 0.001855 | 3.7E-12 | 360895 | 8.11402E-05 | 29.29 |
| rs12521638 | 5 | 166458770 | G | A | 0.447056 | 0.010077 | 0.001838 | 4.2E-08 | 360895 | 5.02047E-05 | 18.12 |
| rs1469249 | 5 | 113837198 | A | G | 0.210879 | -0.01297 | 0.002248 | 8E-09 | 360895 | 5.59645E-05 | 20.20 |
| rs2220599 | 5 | 7378854 | G | C | 0.36947 | 0.012313 | 0.001897 | 8.5E-11 | 360895 | 7.06341E-05 | 25.49 |
| rs246723 | 5 | 140519166 | G | A | 0.590096 | -0.01067 | 0.001871 | 1.2E-08 | 360895 | 5.50442E-05 | 19.87 |
| rs34238696 | 5 | 161356241 | G | A | 0.106778 | -0.01682 | 0.00296 | 1.3E-08 | 360895 | 5.39407E-05 | 19.47 |
| rs4704043 | 5 | 72159179 | T | C | 0.713608 | 0.011608 | 0.002018 | 8.9E-09 | 360895 | 5.50783E-05 | 19.88 |
| rs6449708 | 5 | 50851575 | C | T | 0.531571 | -0.01058 | 0.001833 | 7.9E-09 | 360895 | 5.57156E-05 | 20.11 |
| rs17789218 | 6 | 100600097 | C | T | 0.245186 | 0.012695 | 0.00212 | 2.1E-09 | 360895 | 5.96509E-05 | 21.53 |
| rs6935828 | 6 | 140811367 | T | C | 0.556131 | 0.010167 | 0.001836 | 3.1E-08 | 360895 | 5.10296E-05 | 18.42 |
| rs707926 | 6 | 31748820 | A | G | 0.150465 | 0.014537 | 0.002548 | 1.2E-08 | 360895 | 5.40245E-05 | 19.50 |
| rs72828532 | 6 | 19065342 | C | T | 0.179279 | 0.015684 | 0.002382 | 4.5E-11 | 360895 | 7.23874E-05 | 26.13 |
| rs72847500 | 6 | 37643909 | C | T | 0.121024 | 0.015805 | 0.002811 | 1.9E-08 | 360895 | 5.31456E-05 | 19.18 |
| rs806795 | 6 | 26205293 | A | G | 0.470383 | 0.010356 | 0.001824 | 1.4E-08 | 360895 | 5.34301E-05 | 19.28 |
| rs9375188 | 6 | 98555272 | T | C | 0.483548 | 0.015852 | 0.001832 | 5E-18 | 360895 | 0.000125509 | 45.30 |
| rs12706626 | 7 | 124531370 | A | G | 0.383718 | 0.010409 | 0.001877 | 2.9E-08 | 360895 | 5.12466E-05 | 18.50 |
| rs17167210 | 7 | 133339343 | A | G | 0.435554 | -0.0114 | 0.001841 | 6E-10 | 360895 | 6.38713E-05 | 23.05 |
| rs17862355 | 7 | 126970135 | G | T | 0.442114 | -0.01121 | 0.001837 | 1E-09 | 360895 | 6.19943E-05 | 22.37 |
| rs13262595 | 8 | 143316970 | G | A | 0.560611 | 0.015632 | 0.001837 | 1.8E-17 | 360895 | 0.00012038 | 43.45 |
| rs113851275 | 9 | 98297220 | A | G | 0.107713 | 0.018611 | 0.002943 | 2.5E-10 | 360895 | 6.65797E-05 | 24.03 |
| rs7020477 | 9 | 116827760 | G | A | 0.266981 | -0.01197 | 0.002067 | 7E-09 | 360895 | 5.61089E-05 | 20.25 |
| rs73578186 | 9 | 126334485 | T | C | 0.323945 | -0.01229 | 0.001962 | 3.7E-10 | 360895 | 6.61759E-05 | 23.88 |
| rs10828248 | 10 | 21824619 | G | A | 0.344788 | 0.010653 | 0.001918 | 2.8E-08 | 360895 | 5.12733E-05 | 18.51 |
| rs147543875 | 10 | 101624164 | T | C | 0.02267 | -0.03632 | 0.006422 | 1.5E-08 | 360895 | 5.84592E-05 | 21.10 |
| rs7904398 | 10 | 67954193 | T | C | 0.503287 | -0.0102 | 0.001828 | 2.4E-08 | 360895 | 5.20188E-05 | 18.77 |
| rs1448355 | 11 | 131286685 | T | C | 0.618488 | 0.012261 | 0.001886 | 8E-11 | 360895 | 7.09461E-05 | 25.61 |
| rs16912540 | 11 | 13271422 | G | A | 0.136623 | -0.01582 | 0.002661 | 2.7E-09 | 360895 | 5.90772E-05 | 21.32 |
| rs2734833 | 11 | 113292920 | A | G | 0.606682 | 0.012375 | 0.001871 | 3.8E-11 | 360895 | 7.30881E-05 | 26.38 |
| rs12820967 | 12 | 38921745 | C | T | 0.322363 | 0.011892 | 0.001967 | 1.5E-09 | 360895 | 6.17828E-05 | 22.30 |
| rs206965 | 12 | 120856332 | C | T | 0.791153 | -0.01294 | 0.002243 | 7.9E-09 | 360895 | 5.5371E-05 | 19.98 |
| rs7968738 | 12 | 90281747 | A | G | 0.263717 | -0.01242 | 0.00208 | 2.4E-09 | 360895 | 5.98886E-05 | 21.61 |
| rs1987942 | 13 | 54004785 | C | T | 0.616935 | -0.01106 | 0.001895 | 5.4E-09 | 360895 | 5.77998E-05 | 20.86 |
| rs1999244 | 13 | 66821625 | T | A | 0.220345 | 0.013066 | 0.002206 | 3.2E-09 | 360895 | 5.86536E-05 | 21.17 |
| rs9537571 | 13 | 57604700 | A | G | 0.095569 | 0.018954 | 0.003107 | 1.1E-09 | 360895 | 6.21034E-05 | 22.41 |
| rs11259902 | 15 | 83886529 | A | C | 0.200416 | 0.012908 | 0.002286 | 1.6E-08 | 360895 | 5.34012E-05 | 19.27 |
| rs11634155 | 15 | 26693096 | C | T | 0.335422 | -0.01161 | 0.001948 | 2.5E-09 | 360895 | 6.01293E-05 | 21.70 |
| rs166835 | 15 | 47716037 | T | C | 0.556188 | -0.01082 | 0.001843 | 4.3E-09 | 360895 | 5.7828E-05 | 20.87 |
| rs56229818 | 15 | 58662232 | C | T | 0.484413 | -0.01012 | 0.001826 | 3E-08 | 360895 | 5.11504E-05 | 18.46 |
| rs3730399 | 16 | 67229019 | G | A | 0.064399 | -0.02279 | 0.003721 | 9.1E-10 | 360895 | 6.25904E-05 | 22.59 |
| rs11652437 | 17 | 79338469 | A | C | 0.323689 | 0.012784 | 0.001978 | 1E-10 | 360895 | 7.15491E-05 | 25.82 |
| rs12946454 | 17 | 43208121 | T | A | 0.268016 | -0.01224 | 0.002065 | 3.1E-09 | 360895 | 5.87518E-05 | 21.20 |
| rs7209653 | 17 | 19882084 | C | T | 0.294886 | -0.0134 | 0.002002 | 2.2E-11 | 360895 | 7.46155E-05 | 26.93 |
| rs1648906 | 18 | 35311651 | A | G | 0.321177 | -0.01104 | 0.001969 | 2.1E-08 | 360895 | 5.31526E-05 | 19.18 |
| rs613872 | 18 | 53210302 | T | G | 0.826663 | -0.01571 | 0.002415 | 7.7E-11 | 360895 | 7.07298E-05 | 25.53 |
| rs58638214 | 19 | 31864938 | T | C | 0.397608 | -0.01283 | 0.001869 | 6.7E-12 | 360895 | 7.88676E-05 | 28.47 |
| rs306755 | 20 | 3099752 | C | T | 0.47539 | 0.010372 | 0.001828 | 1.4E-08 | 360895 | 5.3661E-05 | 19.37 |
| rs6028090 | 20 | 59856465 | A | G | 0.55468 | 0.012534 | 0.001851 | 1.3E-11 | 360895 | 7.7605E-05 | 28.01 |
| rs136553 | 22 | 27255675 | T | C | 0.376531 | 0.011311 | 0.001885 | 2E-09 | 360895 | 6.00654E-05 | 21.68 |
| rs7288455 | 22 | 39966547 | G | A | 0.566933 | -0.01055 | 0.001846 | 1.1E-08 | 360895 | 5.46353E-05 | 19.72 |

SNP: single nucleotide polymorphism; EA: effect allele; OA: other allele; SE: standard error; R2: proportion of phenotypic variance explained by SNPs.

**Supplementary Table 10. Main characteristics of SNPs to instrument weekly usage of mobile phone in last 3 months.**

| **SNP** | **Chr** | **Position** | **EA** | **OA** | **EAF** | **Beta** | **SE** | **P value** | **Sample size** | **R^2^** | **F** |
| --- | --- | --- | --- | --- | --- | --- | --- | --- | --- | --- | --- |
| rs4322261 | 1 | 98405856 | A | G | 0.836102 | -0.02261 | 0.003981 | 1.3E-08 | 386626 | 0.00014 | 54.17 |
| rs4678803 | 3 | 35775327 | A | G | 0.283722 | 0.022612 | 0.003269 | 4.6E-12 | 386626 | 0.000208 | 80.36 |
| rs247584 | 5 | 88684376 | C | A | 0.633091 | 0.017035 | 0.00305 | 2.3E-08 | 386626 | 0.000135 | 52.13 |
| rs6452980 | 5 | 72689768 | G | A | 0.923657 | 0.033176 | 0.00562 | 3.6E-09 | 386626 | 0.000155 | 60.02 |
| rs2653347 | 6 | 55136617 | T | A | 0.786766 | -0.02039 | 0.003597 | 1.4E-08 | 386626 | 0.000139 | 53.92 |
| rs7783012 | 7 | 114116881 | A | G | 0.591405 | 0.019964 | 0.003003 | 3E-11 | 386626 | 0.000193 | 74.49 |
| rs4638225 | 10 | 118676964 | A | G | 0.249277 | 0.024348 | 0.003418 | 1.1E-12 | 386626 | 0.000222 | 85.80 |
| rs1533053 | 11 | 113552612 | G | C | 0.640038 | 0.018217 | 0.003069 | 2.9E-09 | 386626 | 0.000153 | 59.13 |
| rs530287 | 11 | 28744256 | C | T | 0.518722 | -0.01727 | 0.002974 | 6.4E-09 | 386626 | 0.000149 | 57.57 |
| rs10161414 | 12 | 75372667 | C | T | 0.31569 | -0.01863 | 0.00318 | 4.6E-09 | 386626 | 0.00015 | 58.01 |
| rs12935426 | 16 | 71739649 | G | A | 0.362205 | -0.01726 | 0.003061 | 1.7E-08 | 386626 | 0.000138 | 53.23 |

SNP: single nucleotide polymorphism; EA: effect allele; OA: other allele; SE: standard error; R2: proportion of phenotypic variance explained by SNPs.

**Supplementary Table 11. Main characteristics of SNPs to instrument job involving heavy manual or physical work.**

| **SNP** | **Chr** | **Position** | **EA** | **OA** | **EAF** | **Beta** | **SE** | **P value** | **Sample size** | **R^2^** | **F** |
| --- | --- | --- | --- | --- | --- | --- | --- | --- | --- | --- | --- |
| rs12089815 | 1 | 91189933 | A | G | 0.548546 | -0.01515 | 0.002407 | 3.1E-10 | 263615 | 0.000114 | 29.96 |
| rs2091329 | 1 | 110042079 | G | A | 0.288157 | -0.01589 | 0.00265 | 2E-09 | 263615 | 0.000104 | 27.31 |
| rs2819348 | 1 | 201884952 | C | T | 0.352247 | 0.014558 | 0.002497 | 5.6E-09 | 263615 | 9.67E-05 | 25.50 |
| rs11678979 | 2 | 100802891 | C | T | 0.271768 | -0.01499 | 0.002706 | 3E-08 | 263615 | 8.9E-05 | 23.46 |
| rs34700731 | 2 | 203360745 | A | G | 0.134891 | 0.019769 | 0.003494 | 1.5E-08 | 263615 | 9.12E-05 | 24.05 |
| rs6544763 | 2 | 44813233 | C | T | 0.660134 | 0.013969 | 0.002526 | 3.2E-08 | 263615 | 8.76E-05 | 23.08 |
| rs11130889 | 3 | 62562748 | G | C | 0.075137 | -0.02492 | 0.004557 | 4.5E-08 | 263615 | 8.63E-05 | 22.76 |
| rs11726786 | 4 | 106120756 | G | T | 0.366352 | 0.016595 | 0.002483 | 2.4E-11 | 263615 | 0.000128 | 33.71 |
| rs2318540 | 4 | 67831581 | G | T | 0.572308 | -0.01428 | 0.002422 | 3.7E-09 | 263615 | 9.99E-05 | 26.33 |
| rs1081158 | 5 | 88004616 | T | C | 0.581826 | 0.016989 | 0.00243 | 2.7E-12 | 263615 | 0.00014 | 37.03 |
| rs56194430 | 5 | 67824690 | T | C | 0.169355 | 0.018854 | 0.003215 | 4.5E-09 | 263615 | 0.0001 | 26.37 |
| rs11756123 | 6 | 152218079 | T | A | 0.634651 | -0.01375 | 0.002487 | 3.2E-08 | 263615 | 8.77E-05 | 23.11 |
| rs4731992 | 7 | 133702097 | G | A | 0.781718 | -0.01704 | 0.002895 | 4E-09 | 263615 | 9.91E-05 | 26.13 |
| rs62511803 | 8 | 115992376 | A | G | 0.140624 | -0.02117 | 0.00344 | 7.5E-10 | 263615 | 0.000108 | 28.56 |
| rs77823953 | 8 | 143524185 | T | C | 0.032671 | -0.03716 | 0.006759 | 3.8E-08 | 263615 | 8.73E-05 | 23.02 |
| rs10820625 | 9 | 99203606 | C | T | 0.220898 | -0.01601 | 0.002885 | 2.9E-08 | 263615 | 8.82E-05 | 23.25 |
| rs7108077 | 11 | 95838005 | G | A | 0.379639 | -0.01344 | 0.002463 | 4.9E-08 | 263615 | 8.51E-05 | 22.43 |
| rs1370059 | 13 | 58523077 | G | A | 0.232046 | 0.018082 | 0.002835 | 1.8E-10 | 263615 | 0.000117 | 30.72 |
| rs3785354 | 16 | 28550667 | T | C | 0.367566 | 0.013582 | 0.002467 | 3.7E-08 | 263615 | 8.58E-05 | 22.61 |
| rs8054111 | 16 | 71990651 | G | A | 0.729849 | -0.01535 | 0.002693 | 1.2E-08 | 263615 | 9.29E-05 | 24.49 |
| rs11663824 | 18 | 50745236 | A | C | 0.396798 | 0.013638 | 0.002448 | 2.5E-08 | 263615 | 8.9E-05 | 23.47 |

SNP: single nucleotide polymorphism; EA: effect allele; OA: other allele; SE: standard error; R2: proportion of phenotypic variance explained by SNPs.
